# Automated calibration of consensus weighted distance-based clustering approaches using sharp

**DOI:** 10.1093/bioinformatics/btad635

**Published:** 2023-10-17

**Authors:** Barbara Bodinier, Dragana Vuckovic, Sabrina Rodrigues, Sarah Filippi, Julien Chiquet, Marc Chadeau-Hyam

**Affiliations:** Department of Epidemiology and Biostatistics, Imperial College London, Norfolk place, London W2 1PG, United Kingdom; Department of Epidemiology and Biostatistics, Imperial College London, Norfolk place, London W2 1PG, United Kingdom; Department of Epidemiology and Biostatistics, Imperial College London, Norfolk place, London W2 1PG, United Kingdom; Department of Mathematics, Imperial College London, London SW7 2RH, United Kingdom; UMR MIA Paris-Saclay, AgroParisTech/INRAE, Palaiseau 91123, France; Department of Epidemiology and Biostatistics, Imperial College London, Norfolk place, London W2 1PG, United Kingdom

## Abstract

**Motivation:**

In consensus clustering, a clustering algorithm is used in combination with a subsampling procedure to detect stable clusters. Previous studies on both simulated and real data suggest that consensus clustering outperforms native algorithms.

**Results:**

We extend here consensus clustering to allow for attribute weighting in the calculation of pairwise distances using existing regularized approaches. We propose a procedure for the calibration of the number of clusters (and regularization parameter) by maximizing the sharp score, a novel stability score calculated directly from consensus clustering outputs, making it extremely computationally competitive. Our simulation study shows better clustering performances of (i) approaches calibrated by maximizing the sharp score compared to existing calibration scores and (ii) weighted compared to unweighted approaches in the presence of features that do not contribute to cluster definition. Application on real gene expression data measured in lung tissue reveals clear clusters corresponding to different lung cancer subtypes.

**Availability and implementation:**

The R package sharp (version ≥1.4.3) is available on CRAN at https://CRAN.R-project.org/package=sharp.

## Graphical Abstract

**Graphical Abstract:**
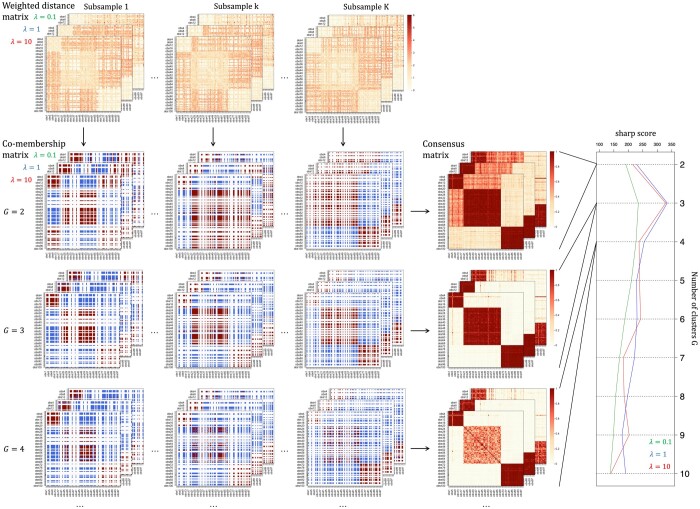
In consensus weighted clustering, the COSA algorithm is applied on multiple subsamples of the data with different regularisation parameters. A co-membership matrix can be obtained by applying a distance-based clustering algorithm (e.g. hierarchical clustering) with a given number of clusters on each of these weighted distance matrices. The consensus matrix is then calculated by aggregating all co-membership matrices over the subsamples for a given regularisation parameter and number of clusters. We propose to calibrate jointly these two hyper-parameters by maximising the sharp score.

## 1 Introduction

Clustering aims at partitioning samples (items) into homogeneous groups with similar features (attributes) (Kaufman and Rousseeuw 2009). These approaches are gaining traction in medicine, e.g. for stratifying patients based on their molecular profiles (Nguyen *et al.* 2017). Multiple clustering algorithms have been proposed, including distance-based clustering, which aims at minimizing the pairwise distances within clusters (compactness) and maximizing the pairwise distances between clusters (separation), and model-based clustering, which makes assumptions about the data generating process and rely on likelihood maximization (Fraley and Raftery 2002). In this article, we focus on the *K* means and distance-based clustering, including hierarchical clustering and Partitioning Around Medoids (PAM), which are computationally efficient and do not require assumptions about the data distribution.

In distance-based clustering, the use of distance metrics defined using vector norms (e.g. Euclidean and Manhattan distances) or correlations (e.g. Pearson’s or Spearman’s correlation) implicitly relies on the assumption that all attributes contribute equally to the clustering (Kampert *et al.* 2017). As a consequence, attributes with low proportions of variance explained by the grouping may dilute the clustering structure and make it more difficult to detect the clusters based on pairwise distances (Friedman and Meulman 2004, Kampert *et al.* 2017). To tackle this issue, regularized approaches have been proposed to generate weighted distances. These include (i) sparse clustering, which estimates attribute-specific weights that are used in the calculation of pairwise distances between items and can be shrunk to exactly zero for attribute selection (Witten and Tibshirani 2010), and (ii) Clustering Objects on Subsets of Attributes (COSA) algorithm, which estimates attribute and item-specific weights (Friedman and Meulman 2004).

The lack of reproducibility or stability of the detected clusters constitutes another challenge. Consensus clustering, where a given clustering algorithm is applied on multiple subsamples of items, has been introduced to generate more stable clusters (Monti *et al.* 2003). The consensus matrix contains pairwise co-membership proportions, which are calculated as the proportions of subsamples where two items are assigned to the same cluster. A distance-based clustering algorithm (e.g. hierarchical clustering) using the consensus matrix as a measure of similarity between the items is then applied to detect the consensus clusters (Monti *et al.* 2003). Consensus clustering has successfully been applied on gene expression data and enabled the identification of stable molecular phenotypes (Hayes *et al.* 2006, Kiselev *et al.* 2017). A simulation study also revealed that consensus clustering is among the best performing clustering approaches when the true number of clusters is known (Pierre-Jean *et al.* 2020). However, the calibration of hyper-parameters hampers its applicability to real-world datasets.

It has been proposed to calibrate the number of clusters using a grid search maximizing the stability of the clustering procedure (Monti *et al.* 2003, Von Luxburg 2010). Existing stability scores include the delta (Δ) score (Monti *et al.* 2003) or Proportion of Ambiguous Clustering (PAC) score (Şenbabaoğlu *et al.* 2014), which measure clustering stability from the estimated distribution of co-membership proportions. Alternatively, it has been proposed to measure the discrepancy between clustering on the full sample and on perturbed data (Nguyen *et al.* 2017). More recently, the Relative Cluster Stability Index (RCSI) has been introduced as a measure of how much the data can be partitioned and is estimated as the difference between the estimated and expected scores (e.g. PAC) under the hypothesis of a single cluster (John *et al.* 2020). The calculation of the RCSI requires the time-consuming application of consensus clustering on several datasets that are simulated from a reference distribution with a single cluster.

In this article, we introduce consensus weighted clustering, where an existing weighted clustering algorithm is applied on multiple subsamples of the items. This approach is controlled by two hyper-parameters, including the regularization parameter and the number of clusters. We propose to calibrate jointly these two hyper-parameters in a grid search maximizing the sharp score, a novel score measuring clustering stability from (weighted) consensus clustering outputs. The sharp score can be computed extremely efficiently and does not incur any further increase in computation time after consensus (weighted) clustering has been run.

In Section 2, we introduce our consensus weighted clustering approach, our calibration procedure and the simulation models implemented. Then, we conduct several simulation studies comparing clustering performances obtained with (i) hierarchical clustering without subsampling, (ii) consensus clustering calibrated as previously proposed (Monti *et al.* 2003, Şenbabaoğlu *et al.* 2014, John *et al.* 2020), and (iii) consensus clustering calibrated by maximizing the sharp score. We also evaluate both the clustering and ranking performances of consensus weighted clustering in simulated and real datasets. Finally, we apply (consensus weighted) clustering to a publicly available transcriptomics dataset including 3312 assayed transcripts measured in 17 normal lung tissues and 46 malignant tumours (Bhattacharjee *et al.* 2001).

## 2 Materials and methods

### 2.1 Consensus weighted clustering

#### 2.1.1 Overview

**Figure 1.**
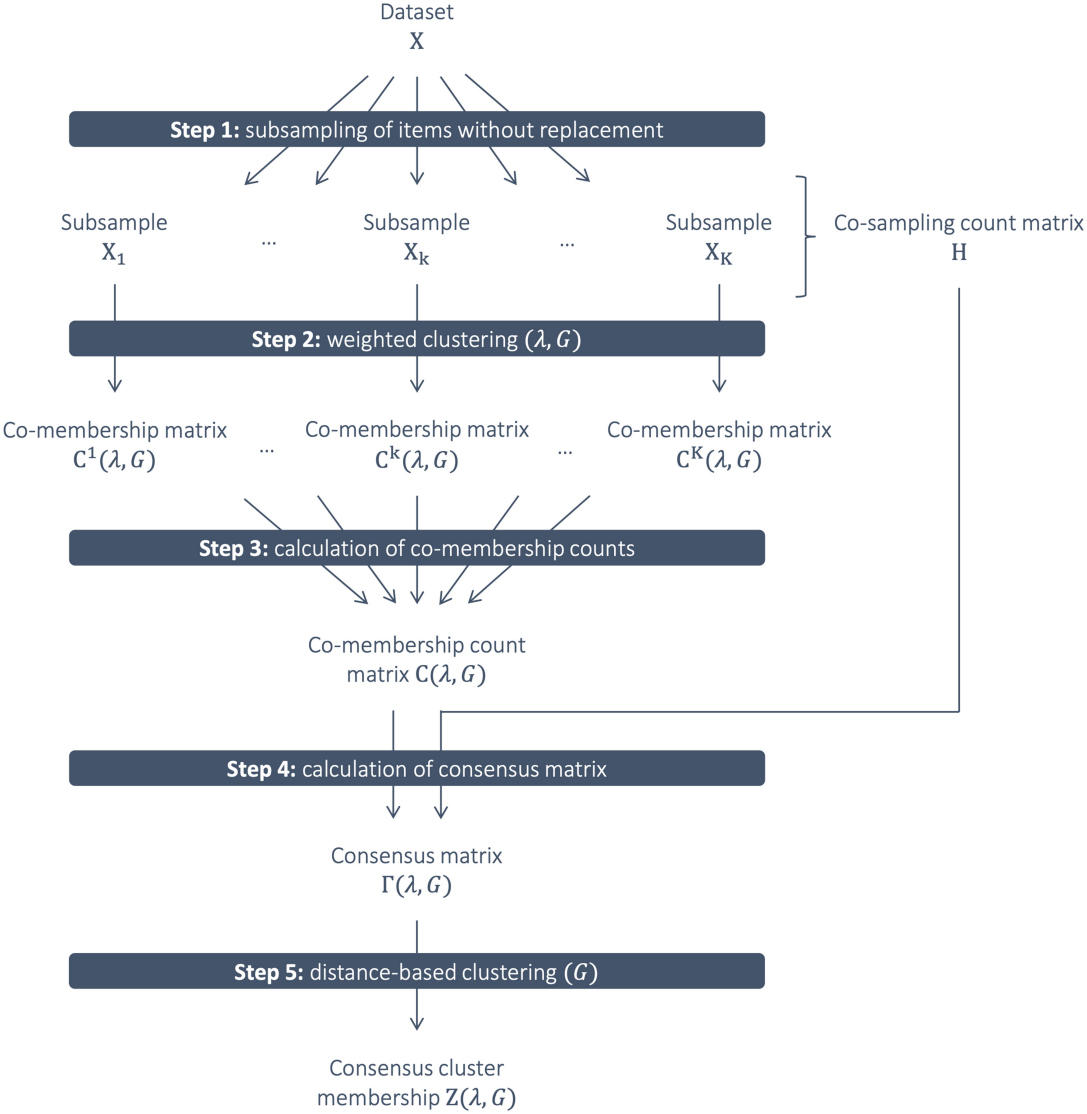
Flowchart describing the five steps of consensus weighted clustering applied on the data matrix *X* with hyper-parameters *λ* and *G.*

The proposed consensus weighted clustering approach can be decomposed into six steps, as described in Fig. 1. Consensus weighted clustering is governed by two hyper-parameters that need to be calibrated: the regularization parameter *λ* for the calculation of weighted distances and the number of clusters *G*.

First, *K* random subsamples of a proportion τ∈[0,1] of the *n* items are drawn without replacement. We use *K *=* *100 and τ=0.5 throughout this article, as in previous studies (John *et al.* 2020, Bodinier *et al.* 2023). The number of subsamples where each pair of items is drawn is stored in the co-sampling count matrix *H*. Second, a (weighted) distance-based clustering algorithm is applied on each of the *K* subsamples to detect *G* clusters. Based on the resulting cluster memberships in each of the *K* subsamples, co-membership matrices indicating which pairs of items belong to the same cluster are calculated. Third, the matrix of co-membership counts C(λ,G) is calculated as the sum over the *K* co-membership matrices. Fourth, the consensus matrix Γ(λ,G) is calculated and contains, for each pair of items, the proportion of co-membership out of the number of subsamples where both items were drawn.

Consensus (weighted) clustering aims at the identification of stable clusters by maximizing the within-cluster and minimizing the between-cluster co-membership proportions obtained over multiple subsamples. In Step 5, a distance-based clustering algorithm is used to generate the *G* stable clusters in Z(λ,G) using the consensus matrix as a measure of similarity.

These steps are described in detail in the following sections.

#### 2.1.2 Weighted distance calculation

We extend the consensus clustering framework by incorporating an algorithm for weighted distance matrix calculation in Step 2 (Fig. 1). We investigate the use of three regularized approaches: sparse *K* means and sparse hierarchical clustering, which both use attribute-specific weights (Witten and Tibshirani 2010), and COSA, which uses attribute- and item-specific weights (Friedman and Meulman 2004, Kampert *et al.* 2017). More details about these methods can be found in the Supplementary Appendix.

#### 2.1.3 Consensus clustering framework

In Step 2 (Fig. 1), a distance-based clustering algorithm is applied on each of the *K* (weighted) distance matrices. The co-membership status $C_{ij}^{k}(\lambda ,G)$ of items *i* and *j* in subsample *k* is defined as:


$$C_{ij}^{k}(\lambda ,G)=\{\begin{array}{c} 1\;\text{if}\;i\neq j\;\text{are both in subsample}\;k\;\text{and are in the same}\\ \text{cluster obtained with parameters}\;\lambda \;\text{and}\;G,\\ 1\;\text{if}\;i=j,\\ 0\;\text{otherwise}. \end{array}$$

The matrix *C* of co-membership counts over the *K* iterations is then calculated in Step 3 (Fig. 1) as:


$$C_{ij}(\lambda ,G)=\sum_{k=1}^{K}C_{ij}^{k}(\lambda ,G).$$

To account for the possibility that items *i* and *j* may not both be included in a given subsample, we define the matrix *H* of co-sampling counts, where *H_ij_* is the number of subsamples that include both items *i* and *j*. By construction, the element *H_ii_* corresponds to the number of subsamples that include item *i*. We consider throughout this article that the same subsamples are used to calculate the co-membership counts for all pairs of parameters (λ,G). That is, the matrix *H* does not depend on *λ* or *G*.

In Step 4 (Fig. 1), entries of the consensus matrix Γ(λ,G) are defined as co-membership proportions calculated over subsamples where both *i* and *j* were included:


$$\Gamma_{ij}(\lambda ,G)=\frac{C_{ij}(\lambda ,G)}{H_{ij}}.$$

As previously proposed (Monti *et al.* 2003, Şenbabaoğlu *et al.* 2014, John *et al.* 2020), the *G* stable clusters in Z(λ,G) are obtained by applying a distance-based clustering algorithm using co-membership proportions as a measure of pairwise similarity (Step 5 in Fig. 1). The use of co-membership proportions instead of the Euclidean distance in the construction of stable clusters may result in the re-assignment of some items (Supplementary Fig. S1).

Note that consensus ‘unweighted’ clustering is recovered by setting *λ *= 0.

### 2.2 Calibration of hyper-parameters

#### 2.2.1 Existing scores

To select the number of clusters *G*, it has been proposed in Monti *et al.* (2003) and Von Luxburg (2010) to compare consensus matrices Γ(G) obtained with different values of *G* and select the one yielding the most stable clustering. Theoretically, the most stable clustering would invariably result in the same partition at all subsampling iterations, yielding a binary consensus matrix. Based on this, the proportion of item pairs with co-membership proportions above a certain threshold *x* was proposed as a measure of stability (Monti *et al.* 2003, Wilkerson and Hayes 2010):


(1) $$CDF_{G}(x)=\frac{1}{n(n-1)/2}\sum_{i<j}\;1_{\Gamma_{ij}(G)\leq x}.$$

Using that metric, Monti *et al.* (2003) propose to determine the number of clusters *G* as the value generating the largest standardized score Δ_*G*_:


$$\Delta_{G}=\{\begin{array}{cc} a_{G} & \text{if}\;G=2.\\ (a_{G}-a_{G-1})/a_{G-1} & \text{if}\;G>2, \end{array}$$

where *a_G_* is the integral of $CDF_{G}(x)$ over [0,1] and increases as co-membership proportions get larger.

Alternatively, the PAC score measures the number of intermediate co-membership proportions (i.e. that are between the lowerbound *x*_1_ and upperbound *x*_2_ of the co-membership proportions) and is defined as (Şenbabaoğlu *et al.* 2014):


$${\text{PAC}}_{G}(x_{1},x_{2})={\text{CDF}}_{G}(x_{2})-{\text{CDF}}_{G}(x_{1}).$$

The number of clusters *G* can then be defined as the one yielding the smallest PAC score (i.e. with the smallest number of intermediate co-membership proportions). The calculation of the PAC score requires the arbitrary choice of two parameters *x*_1_ and *x*_2_. Default values of $x_{1}=0.1$ and $x_{2}=0.9$ were recommended (Şenbabaoğlu *et al.* 2014).

A score measuring the discrepancy between clustering on the full sample and on perturbed datasets has been proposed to choose the number of clusters in the Perturbation clustering for data INtegration and disease Subtyping (PINS) algorithm (Nguyen *et al.* 2017, 2019). The discrepancy score measures clustering stability as the area under the curve of the Cumulative Distribution Function of the difference between the co-membership matrix obtained by applying the clustering algorithm on the full sample and the consensus matrix.

More recently, a Monte Carlo reference-based technique was proposed and implemented in the R package M3C (John *et al.* 2020). The approach is based on the simulation of multiple (package default is 25) datasets from a reference distribution with a single cluster while keeping a similar attribute correlation structure (Tibshirani *et al.* 2001). The number of clusters is then calibrated by maximizing the RCSI of the PAC score or entropy (Zhao *et al.* 2010, John *et al.* 2020) measuring the difference between observed and simulated scores.

#### 2.2.2 The sharp score: a new stability score

In this section, we introduce the sharp score *S_c_* measuring clustering stability and used for the joint calibration of hyper-parameter(s). The sharp score *S_c_* is calculated using the matrix C(λ,G) of co-membership counts, the matrix *H* of co-sampling counts and the consensus clusters Z(λ,G), which are all outputs of consensus clustering (Fig. 1). The co-membership count matrix $C(\hat{\lambda },\hat{G})$ and consensus clusters $Z(\hat{\lambda },\hat{G})$ obtained with the calibrated number of clusters $\hat{G}$ and penalty parameter $\hat{\lambda }$ (if weighted) maximize the score *S_c_*.

**Figure 2.**
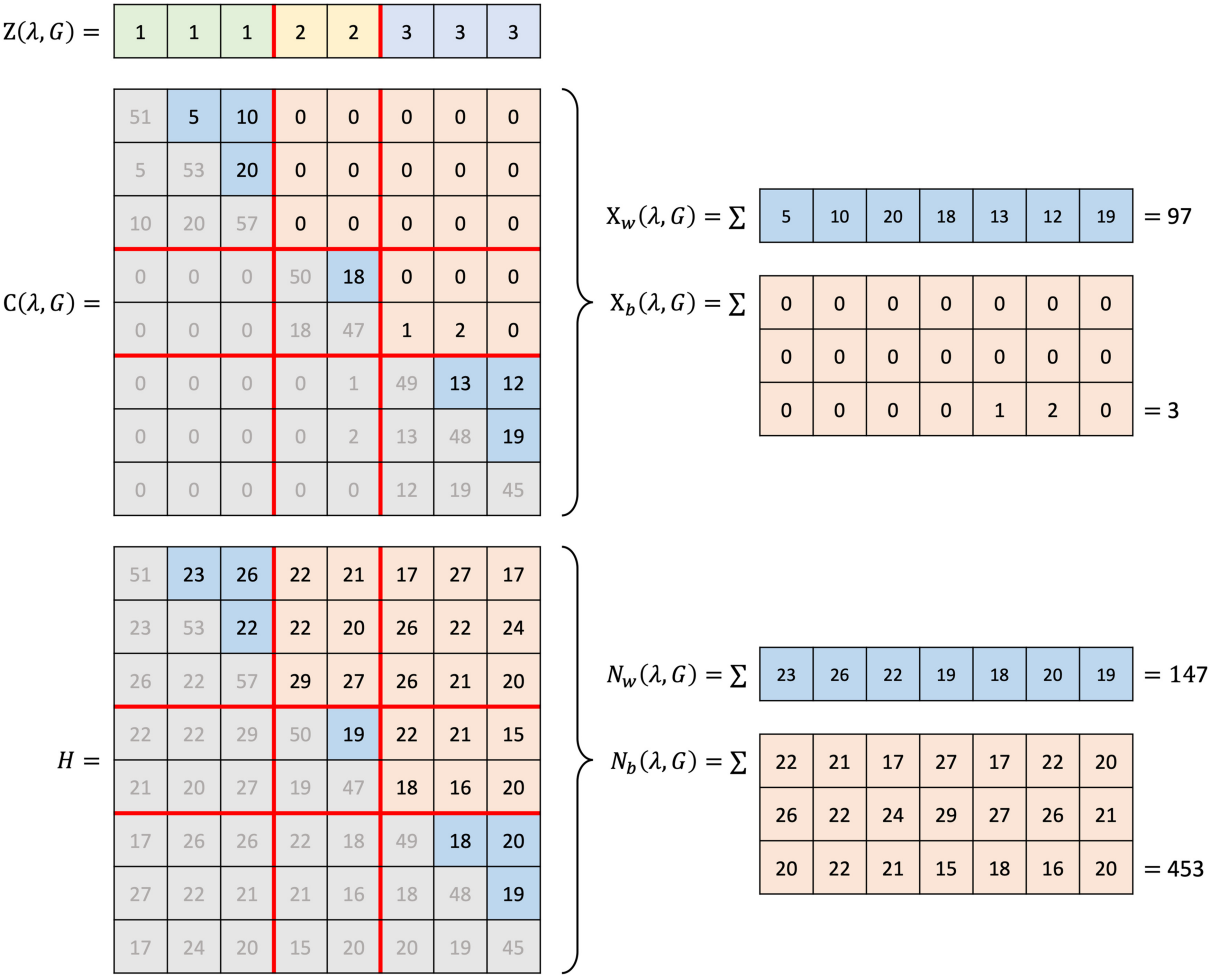
Illustration of the calculation of the quantities $X_{w}(\lambda ,G),\;X_{b}(\lambda ,G),\;N_{w}(\lambda ,G)$, and $N_{b}(\lambda ,G)$ given the consensus clustering outputs Z(λ,G), C(λ,G), and *H*. The blue entries indicate the ‘within’ elements and the orange entries are ‘between’ elements.

To calculate the sharp score for a given pair of hyper-parameters (λ,G), the N=n×(n−1)/2 item pairs are first classified as (i) ‘within’ elements if the two items belong to the same consensus cluster as defined in Z(λ,G) or (ii) ‘between’ elements if the two items belong to different consensus clusters. This is illustrated in Fig. 2, where the blue and orange entries of the co-membership count matrix C(λ,G) and co-sampling count matrix *H* correspond to the within and between elements, respectively.

We introduce the integers $X_{w}(\lambda ,G),\;X_{b}(\lambda ,G),\;N_{w}(\lambda ,G)$ and $N_{b}(\lambda ,G)$ computed as follows (Fig. 2):


(2) $$X_{w}(\lambda ,G)=\sum_{i<j}C_{ij}(\lambda ,G)\;1_{Z_{i}(\lambda ,G)=Z_{j}(\lambda ,G)},$$

(3) $$X_{b}(\lambda ,G)=\sum_{i<j}C_{ij}(\lambda ,G)\;1_{Z_{i}(\lambda ,G)\neq Z_{j}(\lambda ,G)},$$

(4) $$N_{w}(\lambda ,G)=\sum_{i<j}H_{ij}\;1_{Z_{i}(\lambda ,G)=Z_{j}(\lambda ,G)},$$

(5) $$N_{b}(\lambda ,G)=\sum_{i<j}H_{ij}\;1_{Z_{i}(\lambda ,G)\neq Z_{j}(\lambda ,G)}.$$

The quantity $X_{w}(\lambda ,G)$ is the total number of co-members obtained over the *K* subsampling iterations that are among the ‘within’ pairs of items (in blue in Fig. 2). The number $N_{w}(\lambda ,G)$ is the total number of ‘within’ pairs of items that are sampled together over the *K* subsampling iterations. Similarly, $X_{b}(\lambda ,G)$ and $N_{b}(\lambda ,G)$ are the total numbers of co-members and of co-sampled pairs, respectively, among the ‘between’ pairs (in orange in Fig. 2).

We make the assumption that the correlation between the co-membership counts $C_{ij}(\lambda ,G)$ is only due to the clustering structure encoded in $Z_{ij}(\lambda ,G)$. It implies that the co-membership counts $C_{ij}(\lambda ,G)$ follow independent binomial distributions, conditionally on the co-sampling counts *H* and consensus clusters Z(λ,G):


$$C_{ij}(\lambda ,G)|H,Z(\lambda ,G)∼B\left(H_{ij},p_{ij}(\lambda ,G)\right).$$

Our sharp score evaluates if the probabilities $p_{ij}(\lambda ,G)$ are larger for pairs of items in the same consensus cluster (*Z_i_*=*Z_j_*) than for pair of items in different consensus clusters ($Z_{i}\neq Z_{j}$). To devise this score, we assume for simplicity that


$$p_{ij}(\lambda ,G)=\{\begin{array}{c} p_{w}(\lambda ,G)\;\text{if}\;Z_{i}=Z_{j}\\ p_{b}(\lambda ,G)\;\;\text{otherwise}. \end{array}.$$

As a consequence, the quantities $X_{w}(\lambda ,G)$ and $X_{b}(\lambda ,G)$ also follow binomial distributions, conditionally on the co-sampling counts in *H*:


$$\begin{array}{c} X_{w}(\lambda ,G)|H,Z(\lambda ,G)∼B\left(N_{w}(\lambda ,G),p_{w}(\lambda ,G)\right)\;\text{and}\;\\ X_{b}(\lambda ,G)|H,Z(\lambda ,G)∼B\left(N_{b}(\lambda ,G),p_{b}(\lambda ,G)\right). \end{array}$$

We consider that a stable clustering is characterized by a probability $p_{w}(\lambda ,G)$ that is larger than $p_{b}(\lambda ,G)$. To measure clustering stability, we then compare the probabilities $p_{w}(\lambda ,G)$ and $p_{b}(\lambda ,G)$ using a two-sample *z* test where the null hypothesis is $p_{w}(\lambda ,G)\leq p_{b}(\lambda ,G)$. The sharp score *S_c_* is defined as the *z* statistic, calculated as:


$$S_{c}(\lambda ,G)=\frac{{\hat{p}}_{w}(\lambda ,G)-{\hat{p}}_{b}(\lambda ,G)}{\sqrt{{\hat{p}}_{0}(\lambda ,G)\left(1-{\hat{p}}_{0}(\lambda ,G)\right)\left(\frac{1}{N_{w}(\lambda ,G)}+\frac{1}{N_{b}(\lambda ,G)}\right)}},$$

where ${\hat{p}}_{w}(\lambda ,G)=\frac{X_{w}(\lambda ,G)}{N_{w}(\lambda ,G)},\;{\hat{p}}_{b}(\lambda ,G)=\frac{X_{b}(\lambda ,G)}{N_{b}(\lambda ,G)}$, and ${\hat{p}}_{0}(\lambda ,G)=\frac{X_{w}(\lambda ,G)+X_{b}(\lambda ,G)}{N_{w}(\lambda ,G)+N_{b}(\lambda ,G)}$.

For large enough $X_{w}(\lambda ,G)$ and $X_{b}(\lambda ,G)$ (typically $N_{w}(\lambda ,G)p_{w}(\lambda ,G)>10$ and $N_{b}(\lambda ,G)p_{b}(\lambda ,G)>10$), the *z* statistic approximately follows a standard Normal distribution (Montgomery and Runger 2010, Casella and Berger 2012). As such, the sharp score $S_{c}(\lambda ,G)$ is comparable across different numbers of clusters *G* and penalty parameters *λ*. The sharp score $S_{c}(\lambda ,G)$ increases with clustering stability.

To illustrate this score, we now consider the extreme situation where the clustering is the most stable. The most stable clustering would result in a binary consensus matrix, or equivalently in a matrix C(λ,G) where (i) ‘within’ elements are the same as in *H* and (ii) ‘between’ elements are all zero. In this extreme situation, the *z* statistic is equal to $z(\lambda ,G)=\sqrt{N_{w}(\lambda ,G)+N_{b}(\lambda ,G)}=\sqrt{N}$. This is the maximum value that the *z* statistic can take, which results in the maximum value of the stability score (see the Supplementary Appendix for proof). In addition, the *z* statistic does not depend on *λ* or *G* in this situation. This is a desirable property as it implies that the most stable clustering (associated with a binary consensus matrix) would result in the same sharp score regardless of the numbers and sizes of clusters.

The effect of the subsampling procedure is accounted for in the definition of the sharp score via the numbers $N_{w}(\lambda ,G)$ and $N_{b}(\lambda ,G)$ used in the estimation of the probabilities ${\hat{p}}_{w}(\lambda ,G),\;{\hat{p}}_{b}(\lambda ,G)$, and ${\hat{p}}_{0}(\lambda ,G)$. The number of subsampling iterations needs to be large enough for each pair of items to be sampled together at least once. In our implementation in the R package sharp, we return a warning message suggesting to increase the number of subsampling iterations if some pairs of items have never been drawn in the same subsample.

#### 2.2.3 Grid search

The number of clusters *G* (and regularization parameter *λ* for weighted clustering) are calibrated by maximizing the sharp score *S_c_* using a grid search algorithm where the consensus matrix and metric measuring the stability are computed for different values of *G* (and *λ*). The calibrated (set of) parameter(s) is the one that maximizes our sharp score.

### 2.3 Simulation models

#### 2.3.1 Gaussian mixture model

We simulate data *X* including *n* items and *p* attributes from a Gaussian mixture, M, where, ∀i∈{1,…,n} (Fraley and Raftery 2002):


(6) $$\begin{array}{cc} Z_{i}\;\;i.i.d. & ∼M\left(1,\kappa \right)\\ X_{i}|Z_{i}\;\text{independent}\; & ∼N_{p}\left(\mu_{Z_{i}},\Sigma \right) \end{array},$$

where *κ* is a vector of length *G* of probabilities that an item belongs to a given cluster, $\mu_{Z_{i}}$ is the mean vector of length *p* for item *i* belonging to cluster *Z_i_*, and Σ is the covariance matrix of size (p×p).

Items belonging to different clusters are generated using the same covariance matrix Σ but different mean vectors $\mu_{Z_{i}}$. The number and size of the simulated clusters is controlled via the number of entries in vector *κ*. We directly use the vector of true cluster membership *Z* as a simulation parameter in our simulations.

#### 2.3.2 Simulation of cluster means *μ_g_*

To control the level of cluster separation and compactness by attribute, we first sample intermediate cluster- and attribute-specific means *η_gj_* for each cluster g∈{1,…,G} and each attribute j∈{1,…,p} from a Gaussian distribution with mean zero and unit variance. The (G×p) intermediate cluster- and attribute-specific means are then stored in the matrix $\tilde{M}$ of size (n×p) such that ${\tilde{M}}_{ij}=\eta_{Z_{i},j}$.

The level of separation between the clusters along attribute *j* is controlled by the expected proportion of explained variance $E_{j}\in [0,1]$. To ensure that the desired proportion of explained variance is reached, we generate the mean *M_ij_* for item *i* along attribute *j* using:


$$M_{ij}=\frac{\sqrt{E_{j}}\times ({\tilde{M}}_{ij}-\frac{1}{n}\sum_{k=1}^{p}{\tilde{M}}_{ij})}{\sqrt{\frac{1}{n-1}\sum_{i=1}^{n}{\left({\tilde{M}}_{ij}-\frac{1}{n}\sum_{k=1}^{n}{\tilde{M}}_{kj}\right)}^{2}}},$$

where *E_j_* is the desired proportion of variance along attribute *j* explained by the simulated clustering.

The *i*-th row of the simulated matrix *M* is the mean vector $\mu_{Z_{i}}$ and can directly be used in the simulation model presented in Equation (6).

The random sampling of cluster means introduces some variability in the level of separation between pairs of clusters along a given attribute (Supplementary Fig. S2). By aggregating information over multiple attributes, the distances calculated for the simulated data are overall lower for pairs of items belonging to the same cluster than for pairs of items belonging to different clusters. The level of separation and compactness of the clusters is controlled by the number of attributes *p* and the proportions of explained variance by attribute $E_{j},j\in \{1,\ldots ,p\}$ (Supplementary Fig. S3).

By chance, we may obtain some clusters that are very well separated from all others, and sets of clusters that are not as well separated (Supplementary Fig. S4) (Rousseeuw 1987). For example, in Supplementary Fig. S4, Cluster 4 (in red) is very compact and well separated from other clusters, as indicated by the large silhouette widths for all cluster members. On the other hand, Clusters 3 and 5 (yellow and green) include items with silhouette widths below zero indicating poor cluster separation. The proposed simulation procedure controls the overall cluster separation through the proportion of variance explained by the grouping structure for each attribute.

#### 2.3.3 Simulation of covariance matrix Σ

We first simulate a correlation matrix $\tilde{\Sigma }$. We consider two simulation scenarios with (i) independent attributes or (ii) groups of correlated attributes. In the first scenario, the identity matrix is used as correlation matrix. In the second scenario, the correlation matrix $\tilde{\Sigma }$ is simulated as previously proposed in the context of graphical modelling (Bodinier *et al.* 2023). Briefly, we (i) simulate the adjacency matrix of a graph with connected components of random subgraphs, (ii) simulate a corresponding precision matrix, (iii) invert it to obtain a covariance matrix, and (iv) compute the correlation matrix $\tilde{\Sigma }$ from the covariance matrix.

To ensure that the proportion of variance explained by the grouping for variable *X_j_* is equal to *E_j_*, the covariance matrix Σ used in Equation (6) is defined as:


$$\Sigma_{ij}=\sqrt{\left(1-E_{i})\times (1-E_{j}\right)}\times {\tilde{\Sigma }}_{ij}.$$

This simulation model has been implemented in the R package fake (version ≥1.4.0), available on CRAN.

### 2.4 Performance metrics

Clustering performance is measured by the Adjusted Rand Index (ARI) to account for the fact that two items may be in the same cluster by chance. The ARI is calculated by comparing the true and estimated co-memberships (Rand 1971, Hubert and Arabie 1985):


$$\text{ARI}=\frac{2\times (TP\times TN-FP\times FN)}{\left(TP+FP)\times (TN+FP)+(TP+FN)\times (TN+FN\right)},$$

where TP is the number of true positives (i.e. true co-members that are in the same reconstructed clusters), TN is the number of true negatives (i.e. pairs of items that are correctly put in different clusters), and FN is the number of false negatives (i.e. true co-members that are in different reconstructed clusters).

Feature selection performance is measured by the *F*_1_-score (Van Rijsbergen 1979):


$$F_{1}=\frac{2\times P\times R}{P+R},$$

where *P* is the precision and *R* is the recall calculated by comparing the sets of attributes contributing to the clustering in the simulation (i.e. such that $E_{j}\neq 0$) and attributes with the highest median weights estimated for weighted distances.

## 3 Results

### 3.1 Simulation study

#### 3.1.1 Outline

In this section, we apply (consensus) clustering to simulated datasets with different numbers of items, attributes, clusters, and levels of separation. Unless specified otherwise, inferences are based on hierarchical clustering with complete linkage applied to the (weighted) Euclidean distance. We use *K *=* *100 subsampling iterations throughout this article. First, we compare the clustering performances of hierarchical clustering and consensus unweighted clustering calibrated using different strategies. Then, we evaluate both the clustering and weighting performances of consensus weighted clustering.

For unweighted clustering, comparisons are conducted using 1000 simulated datasets with *p *=* *10 attributes and *n *=* *150 items allocated to $G^{*}=5$ clusters of sizes $N_{1}=20,\;N_{2}=50,\;N_{3}=30,\;N_{4}=10,\;N_{5}=40$. The *p* attributes all have the same proportion *E* of explained variance by the clustering. Different levels of cluster separation are investigated with *E* ranging from 0.4 to 0.6. Sensitivity analyses include different numbers and sizes of clusters.

For weighted clustering, a total of *p *=* *100 independent attributes are simulated, of which $q^{*}=20$ have non-zero proportions of explained variance (*E *=* *0.6). Sensitivity analyses use different numbers of items, attributes, clusters, different proportions of explained attribute variances by the clustering, and/or groups of correlated attributes.

#### 3.1.2 Comparison of calibration scores

**Figure 3.**
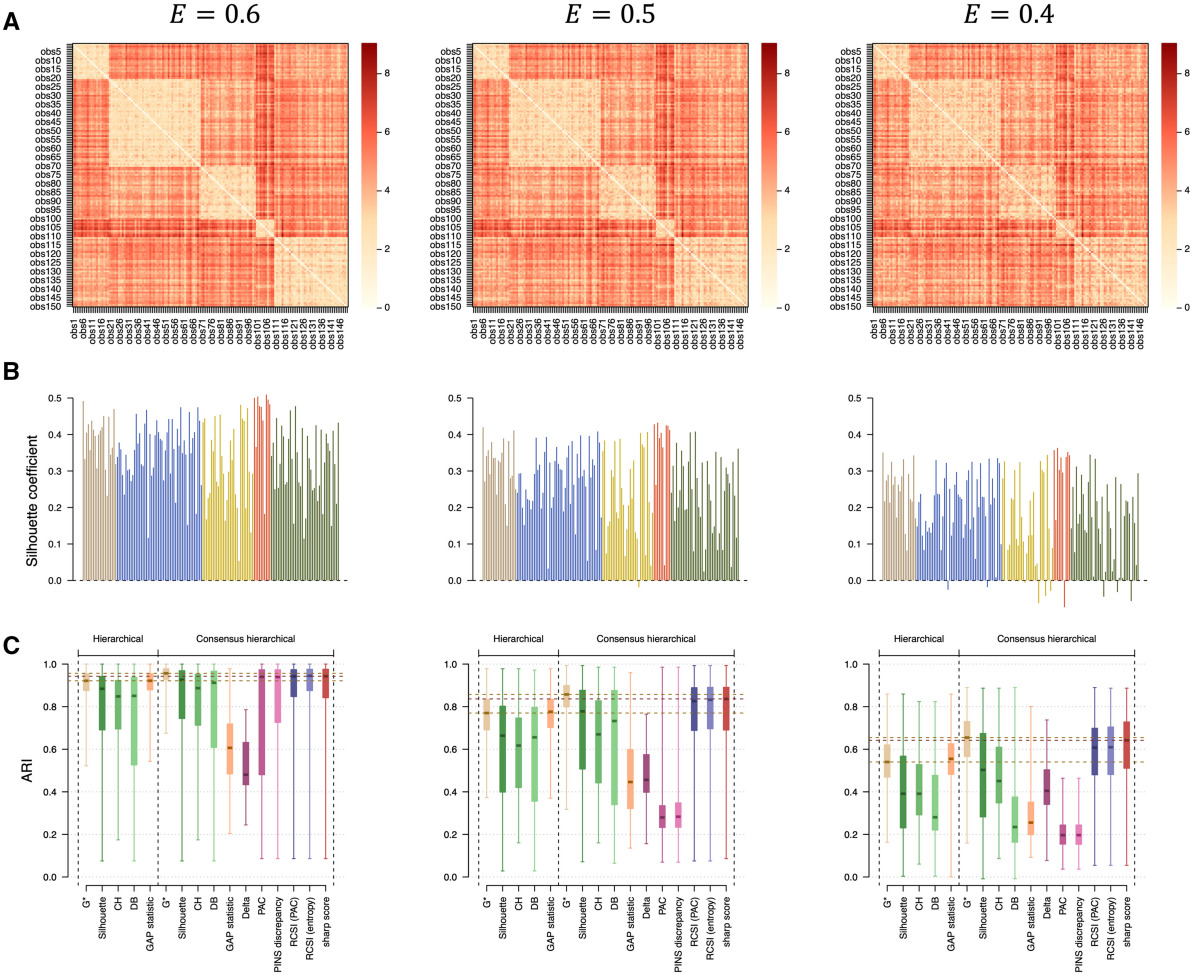
Comparison of clustering performances of (consensus) hierarchical clustering with different calibration strategies from *N *=* *1000 simulated datasets corresponding to different levels of cluster separation. We simulate *N *=* *1000 datasets with *n *=* *150 items split into $G^{*}=5$ clusters such that $N_{1}=20,\;N_{2}=50,\;N_{3}=30,\;N_{4}=10,\;N_{5}=40$ across *p *=* *10 features, each with a proportion of explained variance of *E *=* *0.6 (left), *E *=* *0.5 (middle,) or *E *=* *0.4 (right). For each scenario, we show a heatmap of Euclidean distances (A) and barplot of silhouette widths for each of the *n *=* *150 items coloured by simulated cluster membership (B) for one simulated dataset. Median, quartiles, minimum, and maximum ARI for hierarchical clustering with the simulated number of clusters ($G^{*}$), or calibrated by maximizing the silhouette, CH, DB, and GAP score, and for consensus hierarchical clustering with $G^{*}$ or calibrated using the silhouette, CH, DB, Δ, PAC, PINS discrepancy, RCSI, and sharp scores are reported (C).

We evaluate the ability of hierarchical and consensus clustering with different calibration strategies to recover the grouping structure in simulated data with different levels of cluster separation (Fig. 3 and Supplementary Fig. S5). As a reference, we report the performances of both clustering models with the number of clusters used for the simulation $G^{*}=5$ and compare them with results based on the calibrated numbers of clusters.

With the true number of clusters $G^{*}$, consensus clustering yields a larger (better) median ARI than a single run of hierarchical clustering in the three settings, with a larger increase in median ARI for weaker levels of cluster separation (Fig. 3). This can be explained by the re-assignment of items (mostly re-attributed to their correct cluster) when using the consensus matrix as a measure of similarity (Supplementary Fig. S1).

For hierarchical clustering, calibration maximizing the GAP statistic (Tibshirani *et al.* 2001) generates better performances than with the silhouette (Rousseeuw 1987, Maechler *et al.* 2022), Calinski–Harabasz (CH) (Caliński and Harabasz 1974), or Davies–Bouldin (DB) (Davies and Bouldin 1979) scores in all three scenarios (Fig. 3).

For consensus clustering, models calibrated by maximizing the GAP or Δ scores perform poorly in the three settings (Fig. 3). We observe good performances of calibration by the PAC score for well separated clusters only (*E *=* *0.6), but the inter-quartile range of the ARI is larger than for other approaches (Supplementary Table S1). Models calibrated using the RCSI and sharp scores generate the best clustering performances in these scenarios and are able to recover the true number of clusters $G^{*}$ for most simulated datasets (Supplementary Table S1 and Supplementary Fig. S6). For weaker cluster separation (*E *=* *0.4), calibration by maximizing the sharp score generates larger median ARI than all other approaches, including the Monte Carlo-based procedures (increase in median ARI of 0.03, Supplementary Table S1). This suggests that our score allows for the detection of more subtle clustering structures. Increasing the number of Monte Carlo sampling iterations from 25 to 100 does not affect the clustering performances of models calibrated using the RCSI scores (Supplementary Table S2).

The estimation of consensus matrices for 2–20 clusters on these simulated datasets took <5 s using 1 CPU and 1 GB memory (Supplementary Table S1 and Supplementary Fig. S7). The time needed to compute the silhouette, CH, DB, Δ, PAC, or sharp scores is less than a second. The GAP statistics were calculated in 1–2 s. The median time to calculate the RCSI scores was above 2 min for *N *=* *25 Monte Carlo sampling iterations (Supplementary Table S1 and Supplementary Fig. S7).

Overall these results suggest that the proposed score performs at least as well as established approaches in terms of median ARI and generates performances that are very close to those of consensus clustering using the true number of clusters $G^{*}$ in all scenarios (difference in median ARI lower than 0.02), for no increase in computation time once consensus matrices are estimated (Supplementary Table S1 and Supplementary Fig. S7). Conclusions are similar when applying these approaches using PAM (Supplementary Fig. S8), *K* means (Supplementary Fig. S9), and for simulated data with different numbers of items (Supplementary Table S3) or clusters (Supplementary Fig. S10).

#### 3.1.3 Comparison of weighting methods

We simulate data with $G^{*}=5$ clusters that can be observed along $q^{*}=20$ of the *p *=* *100 attributes (i.e. with non-zero proportion of explained variance by the grouping). We use implementations in the R packages rCOSA for COSA and sparcl for hierarchical or *K* means sparse clustering.

**Figure 4.**
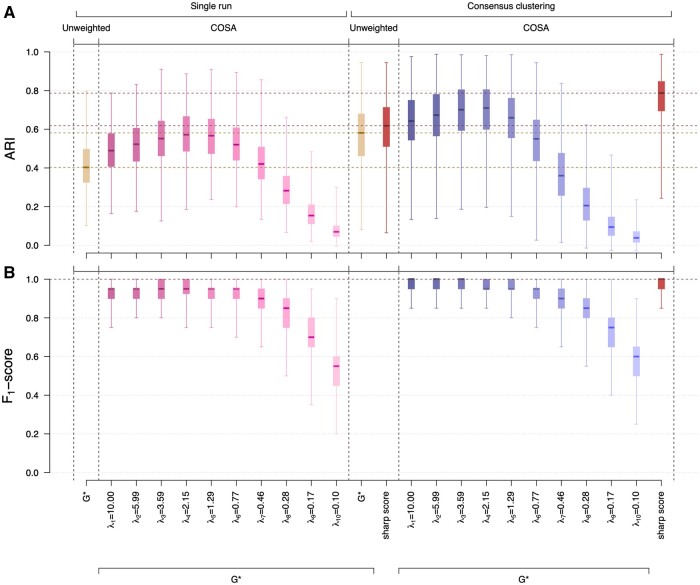
Comparison of clustering performances by the ARI (A) and of the attribute weighting (when applicable) by the *F*_1_-score (B) for hierarchical clustering using unweighted or COSA Euclidean distances and consensus hierarchical clustering using unweighted or COSA Euclidean distances. Performances are evaluated for *N *=* *1000 simulated datasets with *n *=* *150 items split into $G^{*}=5$ clusters including $N_{1}=20,\;N_{2}=50,\;N_{3}=30,\;N_{4}=10,\;N_{5}=40$ items, respectively. The clustering structure is supported by $q^{*}=20$ of the *p *=* *100 attributes with non-zero proportion of explained variance (*E *=* *0.5). Hierarchical clustering is conducted using the true number of clusters $G^{*}$ (in beige or pink). For consensus unweighted clustering, we use the true number of clusters $G^{*}$ (in beige) or calibrated number of clusters by maximizing the sharp score (in red). For consensus weighted clustering, we (i) fix the number of clusters to $G^{*}$ and consider 10 different values of *λ* (in blue), or (ii) jointly calibrate the number of clusters and the penalty parameter using our sharp score (in red). For weighted clustering, the *F*_1_-score measures weighting performance by comparing the sets of (i) the 20 features with highest weights, and (ii) the 20 features supporting the clustering in the simulation (B).

Using the true number of clusters $G^{*}=5$, we observe an increase in clustering performance when introducing weighting in the algorithm with a median ARI of 0.62 for (unweighted) consensus clustering compared to 0.71 for the best regularization parameter using COSA (Fig. 4A and Supplementary Table S4).

Joint calibration of the number of clusters *G* and regularization parameter *λ* using our sharp score yields the highest median ARI at 0.79 (Fig. 4A and Supplementary Table S4). The COSA algorithm does not inherently perform feature selection, but we evaluate here its ability to give larger weights to the contributing features using the *F*_1_-score measuring the selection performance when considering the $q^{*}=20$ features with the largest median weights as selected. The median *F*_1_-score is very close to one, suggesting that the $q^{*}=20$ features with larger median weights are almost always the ones used in the simulation model (Fig. 4B).

Similarly, the consensus sparse *K* means calibrated by maximizing our sharp score yields a better median ARI than consensus unweighted clustering and reaches performances that are very close to the ones observed with the true number of clusters and the best regularization (Supplementary Fig. S11A). In this simple simulation scenario, approaches based on *K* means clustering perform overall better than the approaches based on hierarchical clustering (Supplementary Fig. S11A). The median *F*_1_-score obtained with the calibrated consensus sparse *K* means is also very close to one (Supplementary Fig. S11B).

When using the sparse hierarchical clustering algorithm, the best clustering performances are as good as those obtained with COSA with the true number of clusters $G^{*}$ (highest median ARI of 0.69, Supplementary Fig. S12A). However, these correspond to models that are not sparse, with a median of *q *=* *92 selected attributes (Supplementary Fig. S12B). The *F*_1_-score is calculated by considering the $q^{*}=20$ features with largest selection proportions as selected. The median *F*_1_-score remains below 0.65 for all values of *λ*, suggesting a poorer ability of sparse hierarchical clustering to give larger weights to the contributing features (Fig. 4B). Calibration of consensus sparse hierarchical clustering using our sharp score yields poor clustering performances, with a median ARI of 0.34 and a median calibrated number of clusters $\hat{G}=3$.

The poor weighting and clustering performances when using sparse hierarchical clustering in the proposed approach are likely due to its underlying assumption that the same set of features equally contribute to the definition of all clusters. As this is, in general, not the case with our simulated data (Supplementary Figs S1 and S3), sparse hierarchical clustering does not seem to be able to detect the relevant attributes. These results are in line with previously reported limitations of the sparse hierarchical clustering algorithm (Kampert *et al.* 2017). Based on these results, we do not recommend the use of sparse hierarchical clustering in the proposed consensus weighted clustering calibrated by maximizing the sharp score.

As an alternative, we consider using iteration-specific regularization parameters calibrated by maximizing the GAP statistic in consensus sparse clustering (Supplementary Figs S11 and S12). When using the true number of clusters, the use of iteration-specific regularization does not increase clustering or selection performance compared to the use of a fixed regularization parameter as proposed (Supplementary Figs S11 and S12).

For comparison, we also report performances obtained with IMPACC (Gan and Allen 2022), another approach for attribute selection in consensus hierarchical clustering based on adaptive sampling. This method requires the choice of a proportion of attributes to sample at each iteration. The best clustering performances using IMPACC are comparable to consensus COSA clustering (median ARI of 0.79) but strongly depend on the choice of the attribute sampling proportion (median ARI ranging from 0.54 to 0.82 when using the true number of clusters, Supplementary Fig. S13).

#### 3.1.4 Performances of consensus COSA clustering

Consensus COSA clustering calibrated by maximizing the sharp score also yields a better ARI than (COSA) hierarchical clustering and consensus unweighted clustering for simulated data with a larger number of items (Supplementary Fig. S14A), a larger number of features (Supplementary Fig. S14B) or very unbalanced clusters (Supplementary Fig. S14C).

Outputs generated by consensus COSA clustering include the estimated cluster membership and the distribution of median feature weights estimated from the *K* COSA models with calibrated regularization parameter *λ*. We observe increasing median weights with the proportion of explained variance by feature, which suggests that median weights appropriately capture attribute contribution (Supplementary Fig. S15). However, the overlapping quartiles of median weights for features with proportions of explained variance below 0.4 indicate that features with weaker contributions may be difficult to disentangle from non-contributing features (Supplementary Fig. S10). Introducing correlation between features does not seem to hamper the weighting performances (Supplementary Fig. S16).

### 3.2 Real data applications

#### 3.2.1 Performances of consensus unweighted clustering

We evaluate the clustering performances obtained with (consensus) hierarchical clustering calibrated with different approaches on five publicly available real datasets, including (i) the iris dataset with measurements of 4 attributes for 150 flowers belonging to 3 different species (Becker 2018), (ii) the seeds dataset with measurements of 7 attributes for 210 kernels belonging to 3 different species of wheat (Dua and Graff 2019), (iii) the wine dataset with 13 chemical measurements for 178 wines from 3 different cultivars (Dua and Graff 2019), (iv) the palmer penguins dataset with measurements of 4 attributes for 342 penguins belonging to 3 different species (Horst *et al.* 2022), and (v) the hawks dataset with 5 measurements for 565 hawks belonging to 3 different species available in the R package Stat2Data.

Consensus clustering calibrated by maximizing the sharp score generates the best clustering performance for two of these five datasets (Supplementary Fig. S17). On the remaining three datasets, our approach is the second best approach after the Delta score (on the seeds dataset), the silhouette and CH (on wine), or the RCSI PAC score (on hawks).

#### 3.2.2 Performances of consensus weighted clustering

We apply (consensus) unweighted or COSA clustering on four real molecular datasets obtained with different technologies, including (i) microarray data with 3312 genes for 63 samples of lung tissue that are either healthy or from tumour of 3 subtypes of lung cancer (Bhattacharjee *et al.* 2001), (ii) bulk RNAseq data with 1000 genes for 199 tumour samples corresponding to 4 cancer types (Weinstein *et al.* 2013, Dua and Graff 2019), (iii) single cell RNAseq with 1000 genes for 600 cells belonging to 6 cell lines (Iram and Tabula Muris Consortium 2018), and (iv) single cell RNAseq with 5000 genes for 90 cells belonging to 6 cell types corresponding to different stages of embryonic development (Yan *et al.* 2013, Kiselev *et al.* 2017). These datasets were prepared as detailed in the Supplementary Methods.

Consensus COSA clustering calibrated by maximizing the sharp score generates the best performance in two of these four datasets (Supplementary Fig. S18). In the other two datasets, consensus COSA clustering reaches the second best performance, after consensus unweighted clustering calibrated using our approach. Similarly, the consensus sparse *K* means reaches the best performances in three of the four datasets and is only outperformed by consensus unweighted clustering in the other dataset (Supplementary Fig. S19). As in the simulation study, performances are much lower with consensus sparse hierarchical clustering (Supplementary Fig. S20). IMPACC generates similar clustering performances as consensus COSA clustering in two of the four datasets but performs poorly in the single cell datasets (Supplementary Fig. S21).

#### 3.2.3 Detailed application on microarray data

In this section, we focus on the public microarray data (*p *=* *3312 attributes) in lung cells from *n *=* *63 participants (Bhattacharjee *et al.* 2001). The samples consist of 17 normal lung specimens and 46 lung tumours, including histologically defined squamous-cell lung carcinoma (*N *=* *20), pulmonary carcinoids (*N *=* *20), and small-cell lung carcinoma (*N *=* *6). We perform clustering to detect groups of individuals based on their molecular profiles.

**Figure 5.**
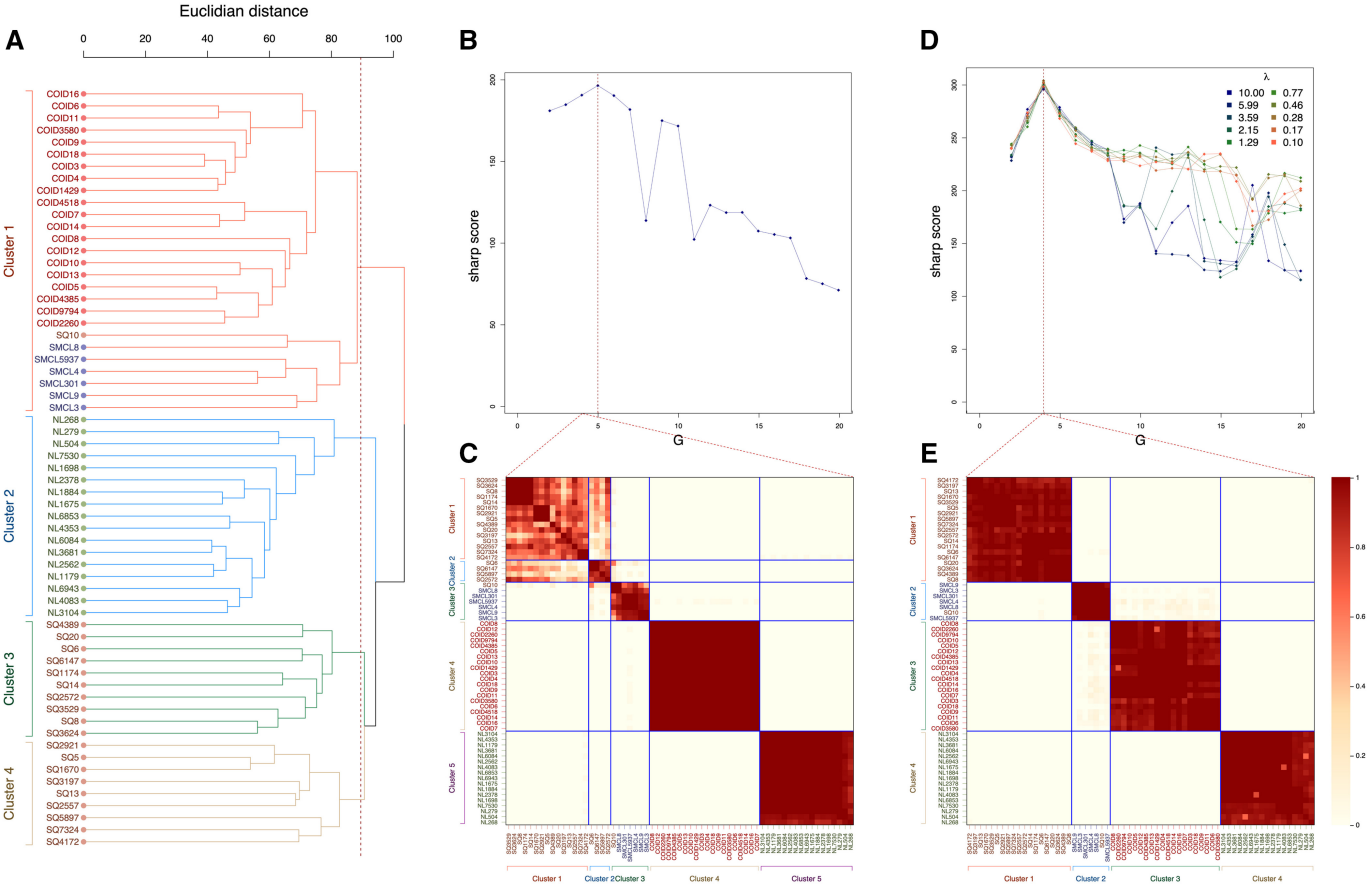
Clustering applications on real transcriptomics data measured in lung tissue. Hierarchical clustering with complete linkage is applied on the Euclidean distance (A). The calibration curve (B) and consensus matrix (C) of consensus clustering using the Euclidean distance are represented. For consensus COSA clustering, we use a grid of 10 regularization parameter in the calibration maximizing the sharp score (D) and report the calibrated consensus matrix (E). Sample names are coloured by type. For each of these three clustering approaches, the four estimated clusters are colour-coded.

First, we apply hierarchical clustering with complete linkage on the Euclidean distances between the *n *=* *63 samples (Fig. 5A). As in the original publication, we observe overall lower distances among samples from healthy lungs or from tumours of the same histological subtype than between these groups. The $G^{*}=4$ clusters obtained from hierarchical clustering include (i) a mixture of *N *=* *20 pulmonary carcinoids, *N *=* *6 small-cell carcinomas, and *N *=* *1 squamous-cell carcinoma in Cluster 1, (ii) all normal lung samples in Cluster 2, and (iii) the squamous-cell carcinomas split over Clusters 3 and 4.

For consensus unweighted clustering, calibration maximizing our score indicates that the most stable clustering is obtained for $\hat{G}=5$ (Fig. 5B). The five stable clusters correspond to (i) *N *=* *19 squamous-cell carcinomas split over Clusters 1 and 2, (ii) all small-cell and the remaining squamous-cell carcinoma in Cluster 3, (iii) all carcinoids in Cluster 4, and (iv) all normal lung tissues in Cluster 5 (Fig. 5C).

For consensus weighted clustering, the calibrated number of clusters is $\hat{G}=4$ and COSA regularization parameter is $\hat{\lambda }=0.28$ (Fig. 5D). The use of weighted distances induces an increase in the sharp score (highest score of 304 compared to 196 in consensus unweighted clustering) that is reflected in the consensus matrix (Fig. 5E). The $\hat{G}=4$ stable clusters in the calibrated consensus weighted clustering correspond to the normal lung samples and the three subtypes of lung cancer, except for one squamous-cell sample that is grouped with the small-cell carcinomas in Cluster 2.

## 4 Discussion

As previously reported (Pierre-Jean *et al.* 2020), we observe better clustering performances with consensus clustering compared to a single run of the underlying (e.g. hierarchical) clustering on both simulated and real data. The use of weighted distances further increases clustering performances in the presence of irrelevant attributes.

Our simulation study and real data applications show that consensus clustering calibrated by maximizing our sharp score generates a median ARI that is at least as good as approaches calibrated using existing scores. Calibration techniques based on cumulative density distributions (PAC and PINS discrepancy scores) only perform well when the clustering structure is extremely strong. Calibration procedures using the consensus and RCSI scores yield similar clustering performances for well separated clusters, but our sharp score allows for the detection of more subtle clustering structures. Furthermore, our sharp score is far less computationally expensive than RCSI scores. Our sharp score is extremely fast to compute (<1 s) from the outputs of consensus clustering with different hyper-parameters, while the RCSI scores require the time-consuming simulation and clustering of multiple datasets and increases computation time by several orders of magnitude.

The assumption that co-membership probabilities are the same for all pairs of items within a given consensus cluster or between a given pair of consensus clusters, respectively, constitutes a potential limitation of our sharp score. This assumption implies that the quantities $X_{w}(\lambda ,G)$ and $X_{b}(\lambda ,G)$ follow binomial distributions, which allows for the use of the *z* test and hence for extremely fast computations. As an alternative, the exact distributions could be recovered using simulations. This alternative may quickly become time consuming due to the resolution required to compare the scores. Despite its underlying assumptions and approximations, the simulation studies indicate very good performances of our sharp score.

More generally, consensus clustering may not perform as well with large numbers of clusters or highly unbalanced clusters as very small clusters may not be represented in the subsamples. The user may consider increasing the subsample size in such situations.

The use of sparse clustering or COSA algorithms in consensus weighting clustering both generate an increase in clustering performance compared to unweighted approaches, for well-chosen regularization parameters. Joint calibration of the number of clusters and regularization parameter in consensus clustering using COSA or sparse *K* means using our sharp score generates some of the best performances. However, the use of sparse hierarchical clustering in consensus weighted clustering calibrated using our sharp score is not recommended as it only detects a subset of the clusters, which leads to overall poor clustering performance. Results from our simulation studies are supported by our applications on real molecular data, where the classes of biological samples are generally better recovered with consensus weighted clustering calibrated using the sharp score. However, this gain in clustering performance comes at the price of an increase in computation time (from 3 s to more than 20 min using COSA or more than 10 h using the sparse *K* means on simulated data with 150 items and 100 features).

The estimated attribute weights in consensus weighted clustering may also help in the biological interpretation of the resulting clusters. Indeed, the median attribute weights (in COSA) or attribute selection proportions (in sparse clustering) could be used to rank the attributes by amount of contribution to the clustering and perform classical gene enrichment analyses (Subramanian *et al.* 2005). We emphasized on results using hierarchical clustering with COSA distances as the use of nearest neighbours in the calculations allows for (i) faster computations, and (ii) the detection of clusters with more complex geometry compared to the sparse *K* means, which is limited to spherical clusters. However, the sparse *K* means has the advantage of performing attribute selection, which may further ease biological interpretation.

Due to the subsampling procedure, the increase in performance with consensus clustering comes at the price of a higher computational burden. Our implementation in the R package sharp allows for parallelization over the subsampling iterations to reduce computation times. The current implementation of consensus clustering does not scale to large numbers of items (e.g. >50 000), which increase both the computation time and memory usage. Extensions potentially using a pre-clustering step are required (Zhang *et al.* 1996, Kaufman and Rousseeuw 2009). Alternatively, future work could consider more efficient subsampling strategies and other optimization strategies beyond grid search (Gan and Allen 2022).

## Supplementary Material

btad635_Supplementary_Data

## Data Availability

Consensus (weighted) clustering and proposed calibration procedure have been implemented in the R package sharp (version ≥1.4.3), available on CRAN at https://CRAN.R-project.org/package=sharp. Simulation models have been implemented in the R package fake (version ≥1.4.0), available on CRAN at https://CRAN.R-project.org/package=fake. The microarray dataset is publicly available and can be downloaded at https://doi.org/10.1073/pnas.191502998. All codes to reproduce the analyses presented in this article are available at https://github.com/barbarabodinier/Consensus_clustering.
