# Supplementary material for: Automated calibration of consensus weighted distance-based clustering approaches using sharp
Source: Bioinformatics. 2023 Oct 17;39(11):btad635. doi: 10.1093/bioinformatics/btad635 (PMC10627366; doi:10.1093/bioinformatics/btad635)
Supplement: btad635_Supplementary_Data [file btad635_supplementary_data.pdf]

Supplementary materials: Automated calibration of consensus weighted distance-based clustering approaches using sharp

Barbara Bodinier[1], Dragana Vuckovic[1], Sabrina Rodrigues[1], Sarah Filippi[2], Julien Chiquet[3] and Marc Chadeau-Hyam[1]

[1] Department of Epidemiology and Biostatistics, School of Public Health, Imperial College London, UK.

[2] Department of Mathematics, Imperial College London, London, UK.

[3] Université Paris-Saclay, AgroParisTech INRAE, UMR MIA, France.

September 24, 2023

1 Supplementary methods

1.1 Sparse clustering

The sparse clustering approach introduced in [1] aims at identifying clusters that are supported by a subset of discriminatory attributes. This is achieved by introducing p attribute-specific weights $w_m, m \in \{1, \ldots, p\}$ in the distance calculations [1]. For given attribute weights w, the sparse clustering distance $d_{ij}^S(w)$ between items i and j can be expressed as:

$$d_{ij}^S(w) = \sum_{m=1}^{p} w_m d_{ijm} \tag{1}$$

where d_{ijm} is the pairwise distance along attribute m.

Attribute weights are estimated by solving a regularised version of the clustering objective function. The sparse hierarchical clustering criterion is [1]:

$$\max_{w,U} \sum_{m=1}^{p} w_m \left[\sum_{i=1}^{n} \sum_{j=1}^{n} \left(d_{ijm} U_{ij} \right) \right], \text{ such that}$$

$$\sum_{ij} U_{ij}^2 \leq 1, ||w||_2^2 \leq 1, ||w||_1 \leq \lambda, w_m \geq 0, \forall m \in \{1, \ldots, p\}$$

where n is the number of items to cluster and U is the overall distance matrix.

In contrast, the sparse K means criterion accounts for cluster membership [1]:

$$\max_{w,C_1,\ldots,C_G} \sum_{m=1}^{p} w_m \Big(\frac{1}{n} \sum_{i=1}^{n} \sum_{j=1}^{n} d_{ijm} - \sum_{g=1}^{G} \frac{1}{n_g} \sum_{i,j \in C_g} d_{ijm} \Big), \text{ such that}$$

$$||w||_2^2 \leq 1, ||w||_1 \leq \lambda, w_m \geq 0, \forall m \in 1, \ldots, p$$

where $C_g, g \in 1, \ldots, G$ is the list of items belonging to cluster g and n_g is the size of cluster g.

The constraint on the ℓ_1-norm of w by a regularisation parameter $\lambda > 1$ induces sparsity, i.e. results in some weights w_m being shrunk to exactly zero. The use of the ℓ_2-norm of w ensures that the clustering is not driven by a single feature. The calibration of the regularisation parameter λ conditionally on the number of clusters can be done using an adapted gap statistic measuring the difference between the observed and expected objectives assuming a single cluster.

1.2 Clustering Objects on Subsets of Attributes

It has been shown that the estimation of a single weight per attribute may result in clusters that are equally spaced along the set of selected features [2, 3]. Clusters that are supported by cluster-specific attributes may therefore be missed. An alternative approach introduces attribute and item specific weights in Clustering Objects on Subsets of Attributes (COSA) [2]. In COSA, the weight matrix W of size $(n \times p)$ is estimated by minimising the sum of weighted distances between each item and its nearest neighbours under a constraint on the weights [2]. Entries of the COSA weighted distance matrix $d^C(W)$ are given by

$$d_{ij}^C(W) = \sum_{m=1}^{p} \max(W_{im}, W_{jm}) d_{ijm}$$

The $(n \times p)$ weights in W are estimated from

$$\min_W \sum_{i=1}^{n} \left[\frac{1}{\sqrt{n}} \sum_{j \in KNN(i)} d_{ij}(W_{i.}) + \lambda \sum_{m=1}^{p} W_{im} \log(W_{im}) \right]$$

where $KNN(i)$ are the $\sqrt{n}$ nearest neighbours of item i, $W_{i.}$ is the ith row of matrix W and $d_{ij}(W_{i.}) = \sum_{m=1}^{p} W_{im} d_{ijm}$.

This optimisation problem is solved approximately using an iterative algorithm. The amount of regularisation is controlled by the parameter λ but does not result in attribute selection.

1.3 Maximum of the sharp score

Recall that the integers $X_w(\lambda, G)$ and $X_b(\lambda, G)$ are the total numbers of co-members in the *within* and *between* pairs, respectively. The integers $N_w(\lambda, G)$ and $N_b(\lambda, G)$ are the total numbers of times each of the *within* and *between* pairs, respectively, are drawn together in the subsamples. For clarity, the λ and G indexing is omitted here. The sharp score S_c can be expressed as a function of X_w, X_b, N_w and N_b:

$$\begin{aligned} S_c &= \frac{\frac{X_w}{N_w} - \frac{X_b}{N_b}}{\sqrt{\left(\frac{X_w+X_b}{N_w+N_b}\right)\left(1 - \frac{X_w+X_b}{N_w+N_b}\right)\left(\frac{1}{N_w} \frac{1}{N_b}\right)}} \\ &= \sqrt{N_w + N_b} \frac{\left(\frac{X_w}{N_w} - \frac{X_b}{N_b}\right)\sqrt{N_w N_b}}{\sqrt{(X_w + X_b)\,(N_w + N_b - X_w - X_b)}} \end{aligned} \quad (2)$$

where $N_w \in \mathbb{N}$, $N_b \in \mathbb{N}$, $X_w \in \{0, \ldots, N_w\}$, $X_b \in \{0, \ldots, N_b\}$, and $(X_w + X_b) \in \{1, \ldots, N_w + N_b - 1\}$.

In this section, we want to find the maximum value of the sharp score S_c. For this, we introduce the real-valued function f of X_w and X_b which is equal to the sharp score when X_w, X_b, N_w and N_b are integers:

$$f(X_w, X_b) = \sqrt{N_w + N_b}\frac{\left(\frac{X_w}{N_w} - \frac{X_b}{N_b}\right)\sqrt{N_w N_b}}{\sqrt{(X_w + X_b)\,(N_w + N_b - X_w - X_b)}} \tag{3}$$

where $N_w > 0$, $N_b > 0$, $X_w \in [0, N_w]$, $X_b \in [0, N_b]$ and $(X_w + X_b) \in [1, \ldots, N_w + N_b - 1]$.

We consider that X_w and X_b belong to these intervals in the remainder of this proof.

The derivatives of f are given by:

$$\frac{\partial f}{\partial X_w}(X_w, X_b) = \frac{\sqrt{N_w N_b}}{\sqrt{(X_w + X_b)(N_w + N_b - X_w - X_b)}}\left[\frac{(N_w + N_b)(X_w + 2X_b + X_b\frac{N_w}{N_b}) - 2(X_w + X_b)(X_b + X_b\frac{N_w}{N_b})}{2N_w(X_w + X_b)(N_w + N_b - X_w - X_b)}\right]$$

$$\frac{\partial f}{\partial X_b}(X_w, X_b) = -\frac{\sqrt{N_w N_b}}{\sqrt{(X_w + X_b)(N_w + N_b - X_w - X_b)}}\left[\frac{(N_w + N_b)(X_b + 2X_w + X_w\frac{N_b}{N_w}) - 2(X_w + X_b)(X_w + X_w\frac{N_b}{N_w})}{2N_b(X_w + X_b)(N_w + N_b - X_w - X_b)}\right]$$

As $N_w > 0$, $N_b > 0$ and $(X_w + X_b) \in [1, \ldots, N_w + N_b - 1]$, we have

$$\frac{\sqrt{N_w N_b}}{\sqrt{(X_w + X_b)(N_w + N_b - X_w - X_b)}} \geq 0 \tag{4}$$

and

$$2N_w(X_w + X_b)(N_w + N_b - X_w - X_b) \geq 0 \tag{5}$$

as well as

$$2N_b(X_w + X_b)(N_w + N_b - X_w - X_b) \geq 0. \tag{6}$$

In addition, we can show that:

- If $X_w \geq X_b\frac{N_w}{N_b}$,

$$
\begin{aligned}
&(N_w+N_b)(X_w+2X_b+X_b\frac{N_w}{N_b})-2\underbrace{(X_w+X_b)}_{<(N_w+N_b)}(X_b+X_b\frac{N_w}{N_b}) \\
&> (N_w+N_b)(X_w+2X_b+X_b\frac{N_w}{N_b})-2(N_w+N_b)(X_b+X_b\frac{N_w}{N_b}) \\
&= (N_w+N_b)(X_w+2X_b+X_b\frac{N_w}{N_b}-2X_b-2X_b\frac{N_w}{N_b}) \\
&= (N_w+N_b)(X_w-X_b\frac{N_w}{N_b}) \\
&\geq 0
\end{aligned}
\tag{7}
$$

as $(X_w+X_b) \leq (N_w+N_b-1)$.

- If $X_w \leq X_b\frac{N_w}{N_b}$,

$$
\begin{aligned}
&(N_w+N_b)(X_w+2X_b+X_b\frac{N_w}{N_b})-2(X_w+X_b)(X_b+X_b\frac{N_w}{N_b}) \\
&\geq (N_w+N_b)(2X_w+2X_b)-2(X_w+X_b)(X_b+X_b\frac{N_w}{N_b}) \\
&= 2(X_w+X_b)(N_w+N_b-\underbrace{X_b}_{\leq N_b}-\underbrace{X_b}_{\leq N_b}\frac{N_w}{N_b}) \\
&\geq 2(X_w+X_b)(N_w+N_b-N_b-N_b\frac{N_w}{N_b}) \\
&= 0
\end{aligned}
\tag{8}
$$

as $X_b \leq N_b$.

Equations 7 and 8 show that

$$(N_w+N_b)(X_w+2X_b+X_b\frac{N_w}{N_b})-2(X_w+X_b)(X_b+X_b\frac{N_w}{N_b}) \geq 0 \tag{9}$$

for any values of X_w and X_b over the intervals where f is defined.

Similarly, we can show that

$$(N_w+N_b)(X_b+2X_w+X_w\frac{N_b}{N_w})-2(X_w+X_b)(X_w+X_w\frac{N_b}{N_w}) \geq 0. \tag{10}$$

Combining Equations 4, 5 and 9, we can show that f is monotonically non-decreasing over X_w as

$$\frac{\partial f}{\partial X_w}(X_w,X_b) \geq 0.$$

Combining Equations 4, 6 and 10, we can show that f is monotonically non-increasing over X_b as

$$\frac{\partial f}{\partial X_b}(X_w, X_b) \leq 0.$$

Hence, the function f is maximised at $X_w = N_w$ and $X_b = 0$, which are the largest and smallest values for X_w and X_b, respectively. The corresponding maximum is:

$$f(X_w = N_w, X_b = N_b) = \sqrt{N_w + N_b}$$

The sharp score can be obtained by applying the function f on integers defined over the same intervals (see Equation 2). As a consequence, the sharp score is also maximised at $X_w = N_w$ and $X_b = 0$, which corresponds to a binary consensus matrix.

To illustrate this result, we represent the values of the sharp score S_c obtained with different values of X_w (x-axis) and X_b (y-axis) in the heatmap below (Figure A). In this example, we used $N_w = 10$ and $N_b = 20$.

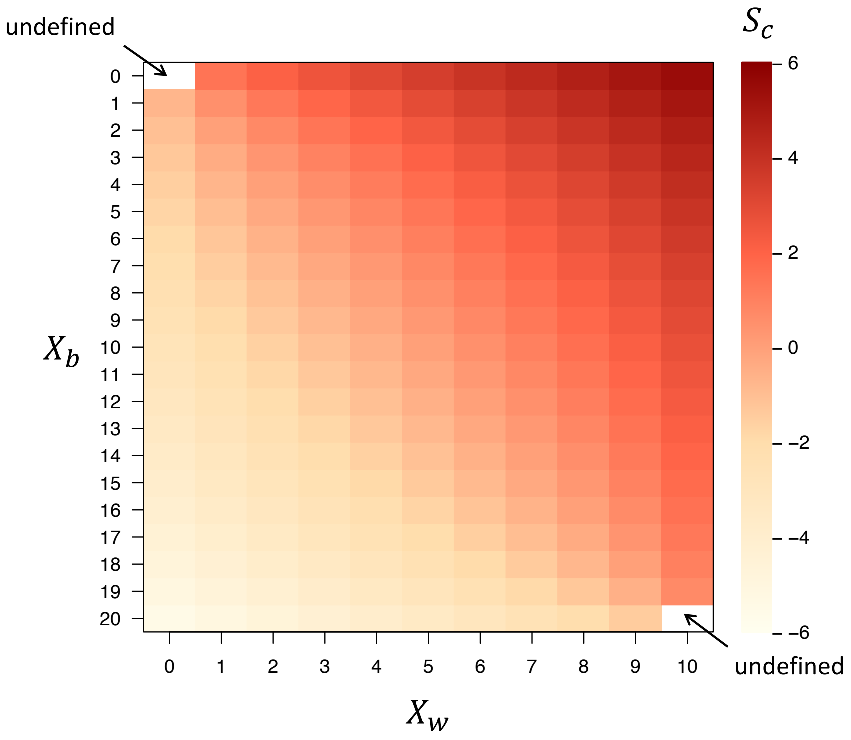

Figure A: Heatmap of the sharp score (colour-coded) obtained with $N_w = 10$ and $N_b = 20$ and different values of X_w (x-axis) and X_b (y-axis).

1.4 Real data preparation

The prepared microarray data was downloaded from the supplementary material of the original publication [4]. Two outliers (AD226 and SQ4) were detected by visual inspection of the data and removed for further analyses. Adenocarcinoma samples were discarded from the data based on the results from the original publication. A total of 17 normal lung tissue samples, along with 20 squamous cell carcinoma, 20 pulmonary carcinoids and 6 small-cell lung carcinoma samples were analysed in the present paper.

The bulk RNAseq data was downloaded from the UCI website (`https://archive.ics.uci.edu/dataset/401/gene+expression+cancer+rna+seq`). The raws counts were transformed as $log_2(x+1)$. The $1,000$ genes with the largest variance were kept for analysis. The breast cancer samples were excluded due to the known molecular heterogeneity within this cancer type. To reduce the computational burden of the experiment, a subset of 199 samples were randomly selected out of the 501 available samples while preserving the proportions of the four cancer types. We analysed 31 colon adenocarcinoma (TCGA-COAD), 58 kidney renal clear cell carcinoma (TCGA-KIRC), 56 lung adenocarcinoma (TCGA-LUAD), and 54 prostate adenocarcinoma samples (TCGA-PRAD) [5].

The Tabula Muris single cell data was loaded using the R package scCCESS [6]. We kept the $1,000$ genes with largest variance for the analysis. The immune cell lines were discarded to use distinct classes. We analysed a total of $1,000$ cells equally distributed over 6 cell types including basal cells, enterocytes of epithelium of large intestine, fibroblasts, keratinocyte stem cells, myeloid cells, and skeletal muscle satellite cells.

The Human single cell data was loaded using the R package SC3 [7,8]. To illustrate the use of our method in a very high dimensional dataset, we kept the $5,000$ genes with the largest variance for the analysis. We considered the 6 cell types provided in the R package SC3 as the true clusters, these correspond to different stages of embryonic development: zygotes, blast cells, two cells, four cells, eight cells and sixteen cells [8].

References

[1] Daniela M Witten and Robert Tibshirani. A framework for feature selection in clustering. *Journal of the American Statistical Association*, 105(490):713–726, 06 2010.

[2] Jerome H. Friedman and Jacqueline J. Meulman. Clustering objects on subsets of attributes (with discussion). *Journal of the Royal Statistical Society: Series B (Statistical Methodology)*, 66(4):815–849, 2004.

[3] Maarten M. Kampert, Jacqueline J. Meulman, and Jerome H. Friedman. rcosa: A software package for clustering objects on subsets of attributes. *Journal of Classification*, 34(3):514–547, 2017.

[4] Arindam Bhattacharjee, William G. Richards, Jane Staunton, Cheng Li, Stefano Monti, Priya Vasa, Christine Ladd, Javad Beheshti, Raphael Bueno, Michael Gillette, Massimo Loda, Griffin Weber, Eugene J. Mark, Eric S. Lander, Wing Wong, Bruce E. Johnson, Todd R. Golub, David J. Sugarbaker, and Matthew Meyerson. Classification of human lung carcinomas by mrna expression profiling reveals distinct adenocarcinoma subclasses. *Proceedings of the National Academy of Sciences*, 98(24):13790–13795, 2001.

[5] John N Weinstein, Eric A Collisson, Gordon B Mills, Kenna R Shaw, Brad A Ozenberger, Kyle Ellrott, Ilya Shmulevich, Chris Sander, and Joshua M Stuart. The cancer genome atlas pan-cancer analysis project. *Nature genetics*, 45(10):1113–1120, 2013.

[6] Tal Iram, Tabula Muris Consortium, et al. Single-cell transcriptomics of 20 mouse organs creates a tabula muris. *Nature*, 562(7727):367–372, 2018.

[7] Vladimir Yu Kiselev, Kristina Kirschner, Michael T Schaub, Tallulah Andrews, Andrew Yiu, Tamir Chandra, Kedar N Natarajan, Wolf Reik, Mauricio Barahona, Anthony R Green, and Martin Hemberg. Sc3: consensus clustering of single-cell rna-seq data. *Nature Methods*, 14(5):483–486, 2017.

[8] Liying Yan, Mingyu Yang, Hongshan Guo, Lu Yang, Jun Wu, Rong Li, Ping Liu, Ying Lian, Xiaoying Zheng, Jie Yan, et al. Single-cell rna-seq profiling of human preimplantation embryos and embryonic stem cells. *Nature structural & molecular biology*, 20(9):1131–1139, 2013.

2 Supplementary figures

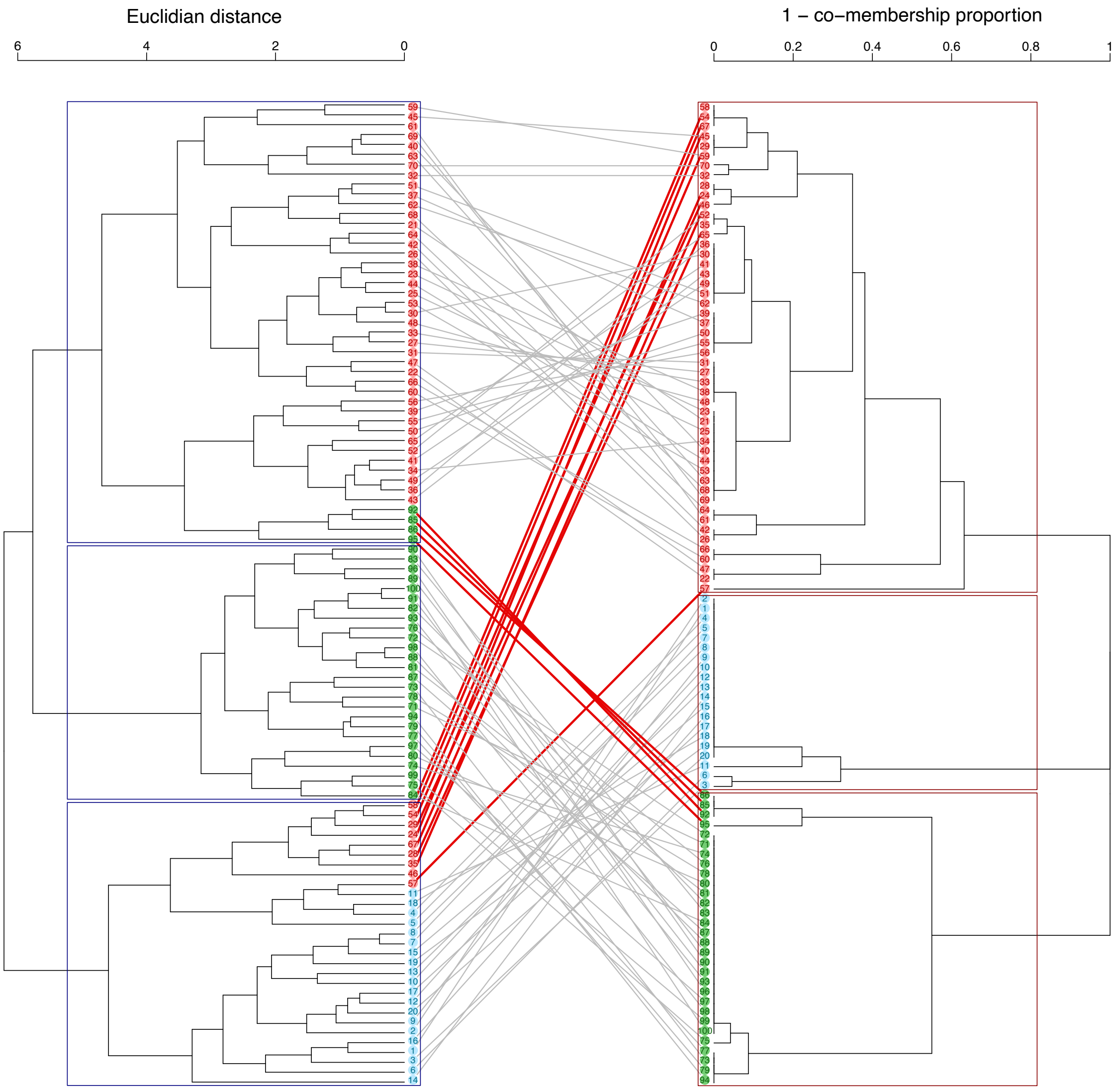

Supplementary Figure 1: Comparison of cluster membership by applying hierarchical clustering with complete linkage using the Euclidean distance (left) or 1 - co-membership proportion (right) as a distance measure. The co-membership proportions are obtained from hierarchical clustering on the Euclidean distances calculated on $K = 100$ subsamples. We represent the dendrograms obtained with the two approaches. The items are coloured by true (simulated) cluster membership. The same items are present in both dendrograms but may be re-ordered due to the change of distance metric. Positions of the same item in the two dendrograms are connected by edges to improve readability. Cluster re-assignment is indicated by red edges.

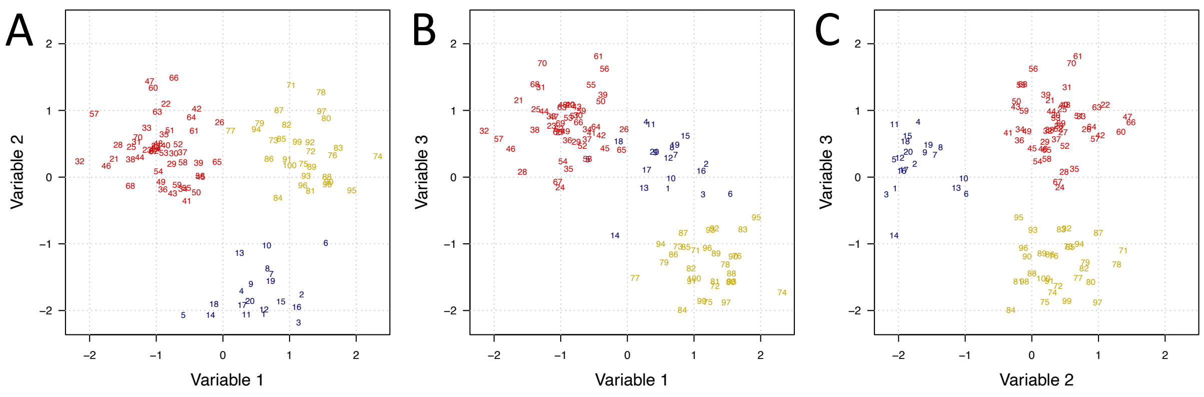

Supplementary Figure 2: Scatter plots for simulated data using $n = 100$ items split into $G^* = 3$ clusters such that $N_1 = 20$ (in blue), $N_2 = 50$ (in red) and $N_3 = 30$ (in orange) across $p = 3$ features with a proportion of explained variance by the grouping structure set to $E = 0.8$ for all features.

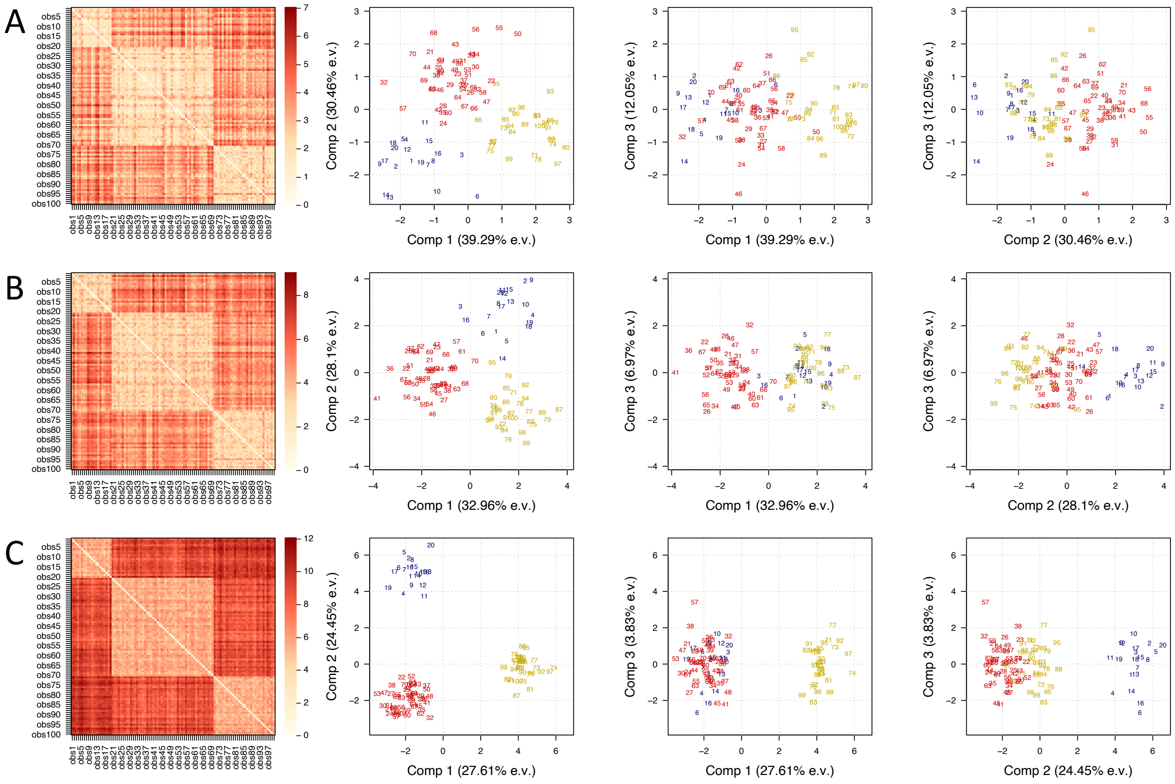

Supplementary Figure 3: Example of simulated data using $n = 100$ items split into $G^* = 3$ clusters such that $N_1 = 20$ (in blue), $N_2 = 50$ (in red) and $N_3 = 30$ (in orange) across $p = 5$ (A), $p = 10$ (B) or $p = 30$ (C) features with a proportion of explained variance by the grouping structure set to $E = 0.5$ for all features. For each dataset, we show the heatmap of Euclidean distances (left) and score plots along the first three principal components of a Principal Component Analysis (right).

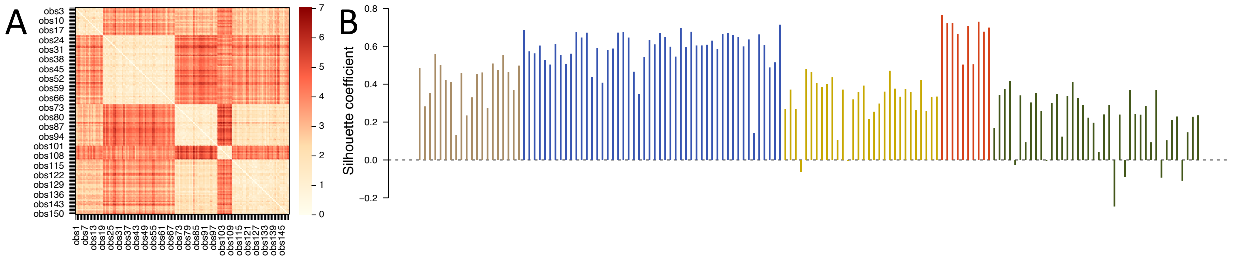

Supplementary Figure 4: Example of simulated data using $n = 150$ items split into $G^* = 5$ clusters such that $N_1 = 20$, $N_2 = 50$, $N_3 = 30$, $N_4 = 10$, $N_5 = 40$ across $p = 5$ features with a proportion of explained variance by the grouping structure set to $E = 0.8$ for all features. We show the heatmap of Euclidean distances (A) and silhouette widths for each of the $n = 150$ items coloured by simulated cluster membership (B).

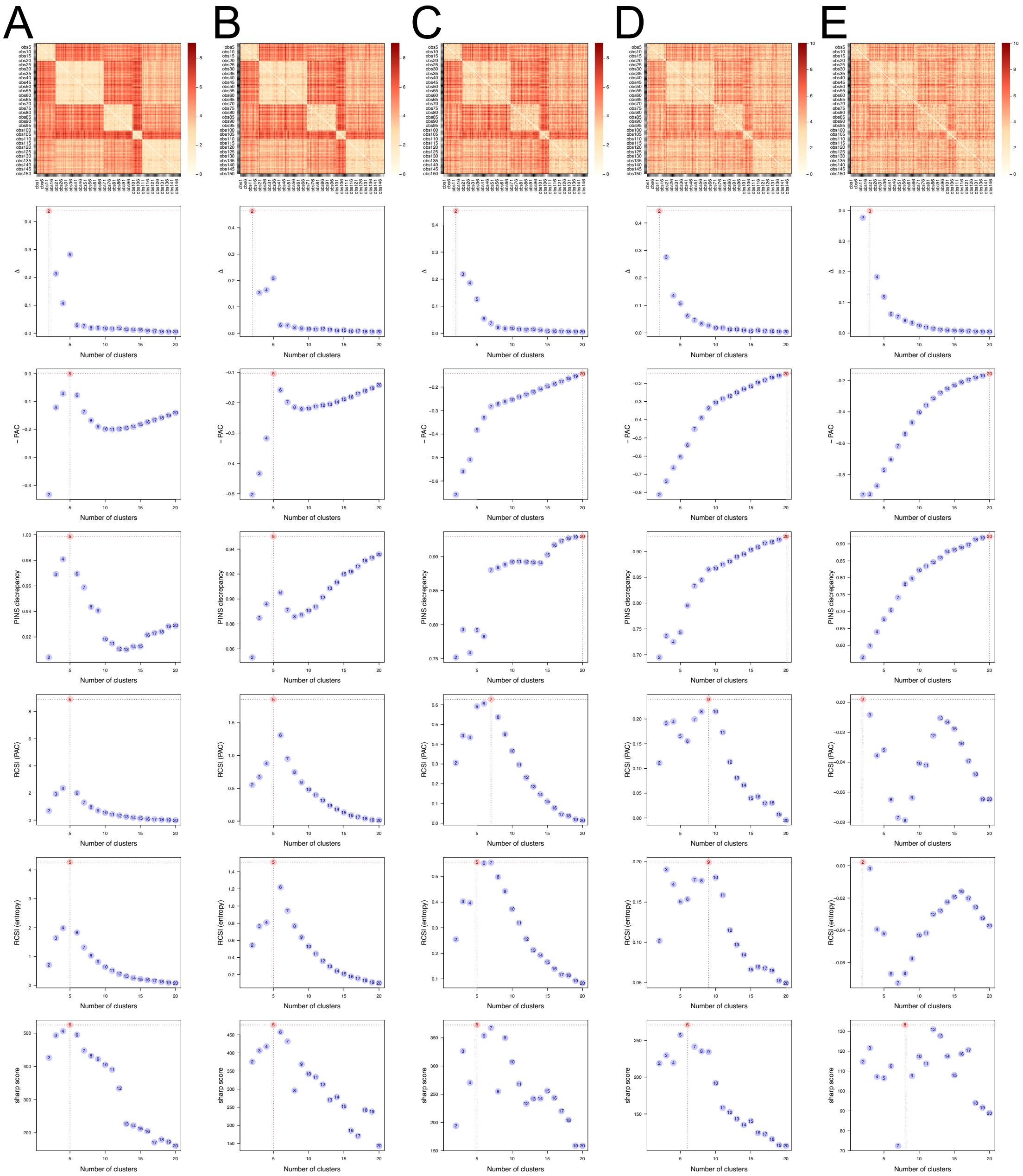

Supplementary Figure 5: Calibration curves using the PAC, Δ, PINS discrepancy, RCSI and sharp scores on simulated examples with different levels of cluster separation and compactness. Consensus clustering was conducted using hierarchical clustering with complete linkage on the Euclidean distances computed on $K = 100$ subsamples. Data is simulated for $n = 150$ items split into $G^* = 5$ clusters such that $N_1 = 20$, $N_2 = 50$, $N_3 = 30$, $N_4 = 10$, $N_5 = 40$ across $p = 10$ features with a proportion of explained variance by the grouping structure set to $E = 0.7$ (A), $E = 0.6$ (B), $E = 0.5$ (C), $E = 0.4$ (D), or $E = 0.3$ (E) for all features. Heatmaps of Euclidean distances for calculated for the simulated data are reported at the top.

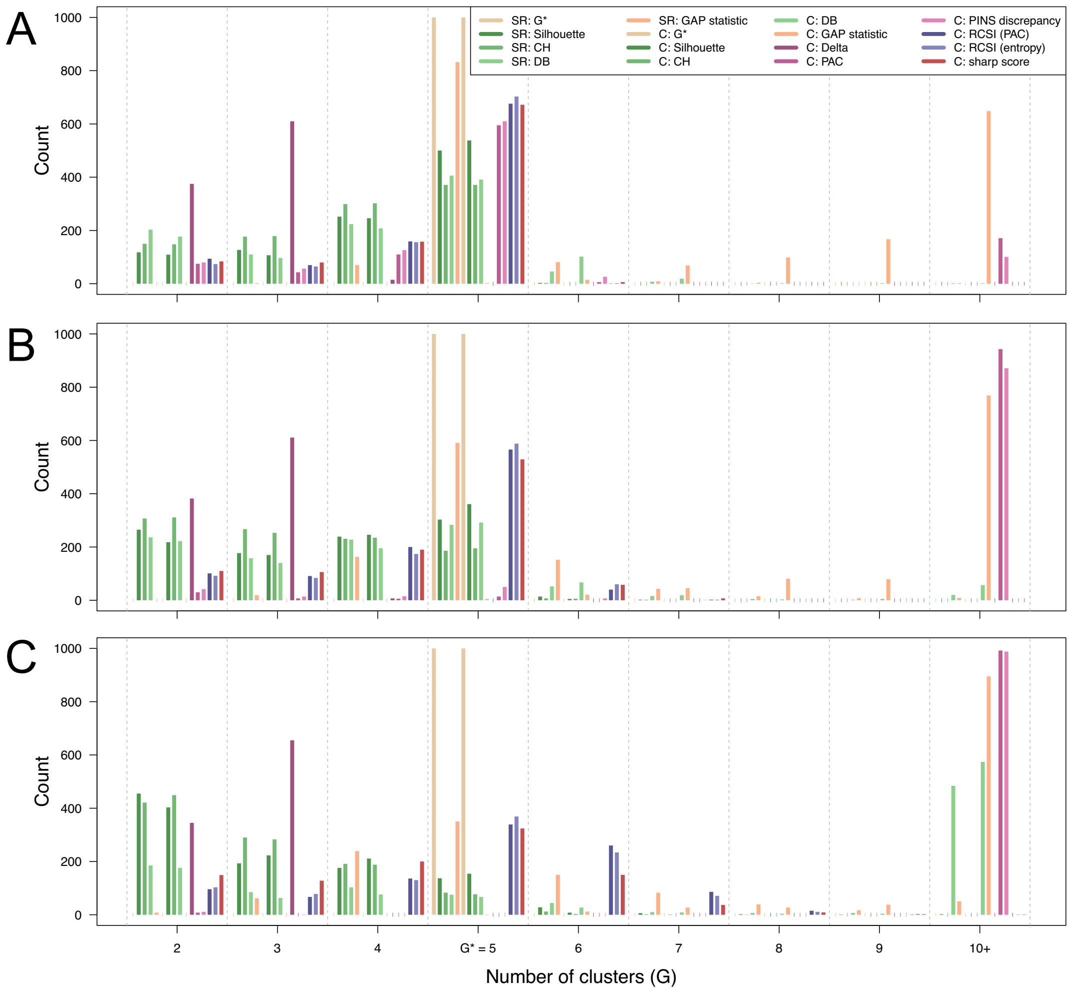

Supplementary Figure 6: Barplot of the counts of simulated datasets for which each calibration method selected a given number of clusters in (consensus) hierarchical clustering. We simulate $N = 1{,}000$ datasets with $n = 150$ items split into $G^* = 5$ clusters such that $N_1 = 20$, $N_2 = 50$, $N_3 = 30$, $N_4 = 10$, $N_5 = 40$ across $p = 10$ features, each with a proportion of explained variance of $E = 0.6$ (A), $E = 0.5$ (B) or $E = 0.4$ (C). We apply a single run of hierarchical clustering (denoted by SR in the legend) or consensus clustering (denoted by C in the legend). For hierarchical clustering, we use the simulated number of clusters (G^*), or calibration maximising the silhouette, Calinski–Harabasz (CH), Davies–Bouldin (DB) or GAP score. For consensus hierarchical clustering, we use G^* or calibration using the silhouette, Calinski–Harabasz (CH), Davies–Bouldin (DB), Δ, PAC, PINS discrepancy, RCSI or sharp scores. The bars are coloured by calibration method. Numbers of clusters ranging from 2 to 9 are showed. Counts for numbers of clusters equal to or greater than 10 are aggregated in the 10+ bars.

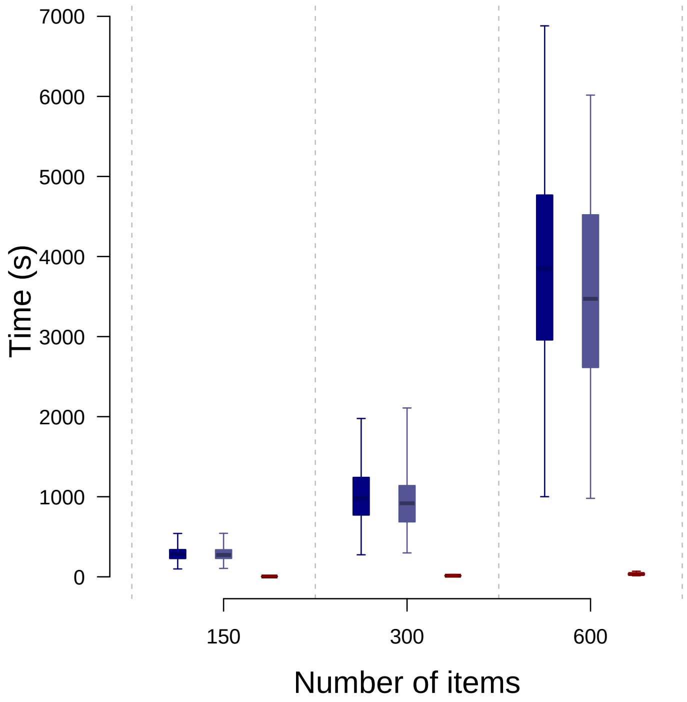

Supplementary Figure 7: Computation times for consensus hierarchical clustering using the RCSI PAC (in dark blue), RCSI entropy (in blue) or sharp score (in red) for different numbers of items. We simulate $N = 1{,}000$ datasets with $n = 150$, $n = 300$, or $n = 600$ items split into $G^* = 5$ clusters of different sizes. We use $p = 10$ attributes with a proportion of explained variance of $E = 0.4$. Median, quartiles, minimum and maximum computation time (in second) are reported for each calibration method and simulation scenario.

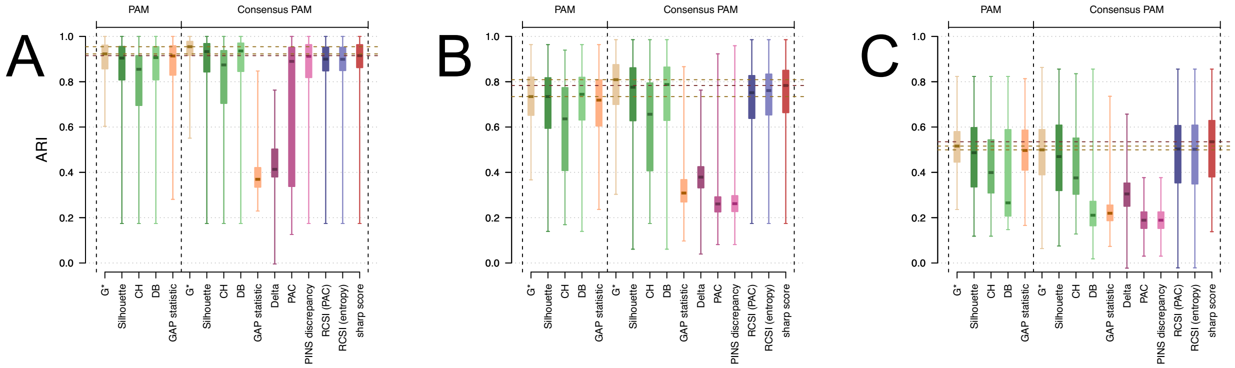

Supplementary Figure 8: Comparison of clustering performances of (consensus) Partitioning Around Medoids (PAM) with different calibration strategies from $N = 1,000$ simulated datasets corresponding to different levels of cluster separation. We simulate $N = 1,000$ datasets with $n = 150$ items split into $G^* = 5$ clusters such that $N_1 = 20$, $N_2 = 50$, $N_3 = 30$, $N_4 = 10$, $N_5 = 40$ across $p = 10$ features, each with a proportion of explained variance of $E = 0.6$ (left), $E = 0.5$ (middle) or $E = 0.4$ (right). Median, quartiles, minimum and maximum Adjusted Rand Index (ARI) for PAM with the simulated number of clusters (G^*), or calibrated by maximising the silhouette and GAP score, and for consensus PAM with G^* or calibrated using the Δ, PAC, PINS discrepancy, RCSI and sharp scores are reported.

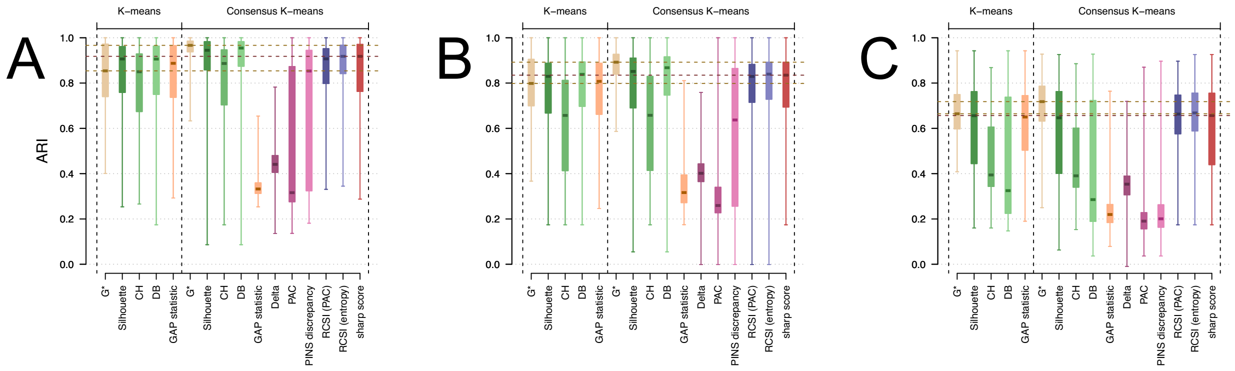

Supplementary Figure 9: Comparison of clustering performances of (consensus) K means with different calibration strategies from $N = 1,000$ simulated datasets corresponding to different levels of cluster separation. We simulate $N = 1,000$ datasets with $n = 150$ items split into $G^* = 5$ clusters such that $N_1 = 20$, $N_2 = 50$, $N_3 = 30$, $N_4 = 10$, $N_5 = 40$ across $p = 10$ features, each with a proportion of explained variance of $E = 0.6$ (left), $E = 0.5$ (middle) or $E = 0.4$ (right). Median, quartiles, minimum and maximum Adjusted Rand Index (ARI) for K means with the simulated number of clusters (G^*), or calibrated by maximising the silhouette and GAP score, and for consensus K means with G^* or calibrated using the Δ, PAC, PINS discrepancy, RCSI and sharp scores are reported.

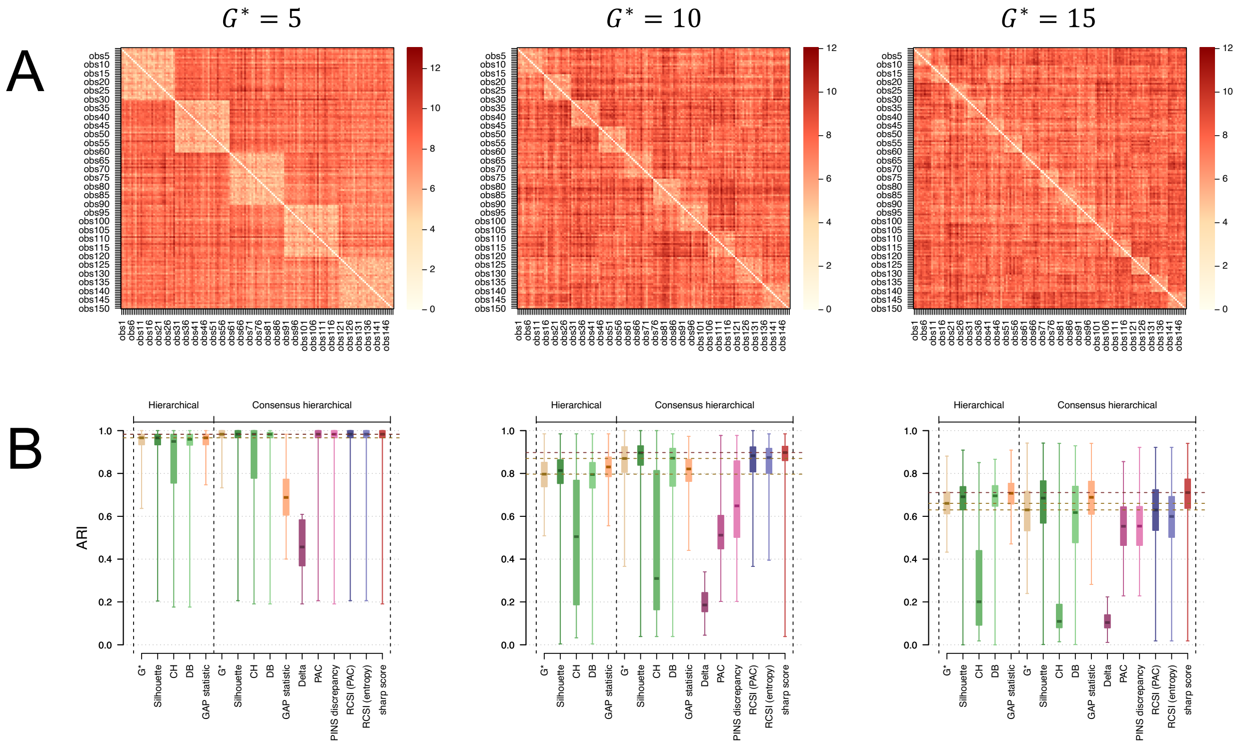

Supplementary Figure 10: Comparison of clustering performances of (consensus) hierarchical clustering with different calibration strategies from $N = 1,000$ simulated datasets corresponding to different numbers of clusters. We simulate $N = 1,000$ datasets with $n = 150$ items split into $G^* = 5$ (left), $G = 10$ (middle), or $G = 15$ (right) clusters of equal sizes across $p = 10$ features, each with a proportion of explained variance of $E = 0.5$. For each scenario, we show a heatmap of Euclidean distances (A). Median, quartiles, minimum and maximum Adjusted Rand Index (ARI) for hierarchical clustering with the simulated number of clusters (G^*), or calibrated by maximising the silhouette and GAP score, and for consensus hierarchical clustering with G^* or calibrated using the Δ, PAC, PINS discrepancy, RCSI and sharp scores are reported (B).

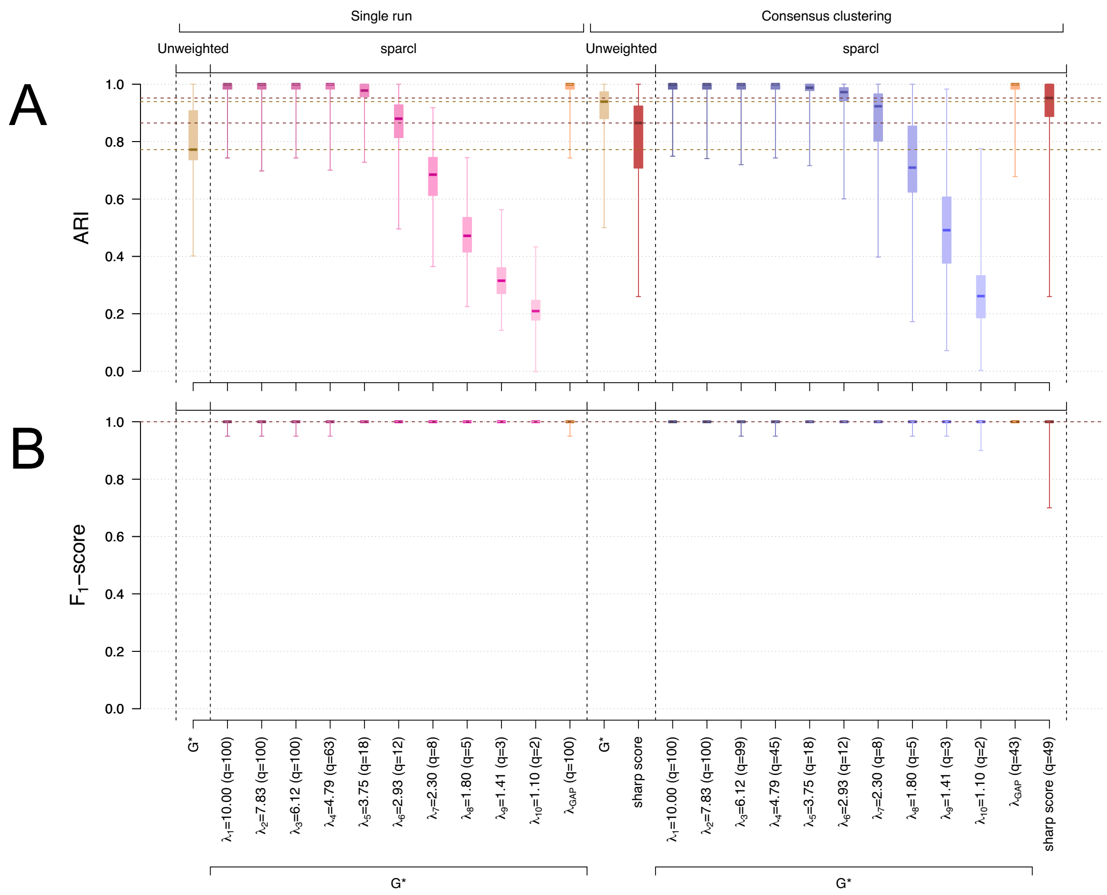

Supplementary Figure 11: Comparison of clustering performances by the Adjusted Rand Index (ARI) (A) and of the attribute weighting (when applicable) by the F_1-score (B) for (consensus) K means or sparse K means. Performances are evaluated for $N = 1,000$ simulated datasets with $n = 150$ items split into $G^* = 5$ clusters including $N_1 = 20$, $N_2 = 50$, $N_3 = 30$, $N_4 = 10$, $N_5 = 40$ items, respectively. The clustering structure is supported by $q^* = 20$ of the $p = 100$ attributes with nonzero proportion of explained variance ($E = 0.5$). K means clustering is conducted using the true number of clusters G^* (in beige, pink or orange). For sparse clustering, we report performances using the true number of clusters G^* and (i) a range of regularisation parameters (in pink), or (ii) with the regularisation parameter calibrated by maximising the GAP statistic (reported as λ_{GAP}, in orange). For consensus unweighted clustering, we use the true number of clusters G^* (in beige) or calibrated number of clusters by maximising the sharp score (in red). For consensus weighted clustering, we (i) fix the number of clusters to G^* and consider ten different values of λ (in blue), (ii) use iteration-specific regularisation parameters calibrated by maximising the GAP statistic (λ_{GAP}, in orange), or (iii) jointly calibrate the number of clusters and the penalty parameter using our sharp score (in red). For weighted

clustering, the F_1-score measures weighting performance by comparing the sets of (i) the 20 features with highest selection proportions, and (ii) the 20 features supporting the clustering in the simulation (B).

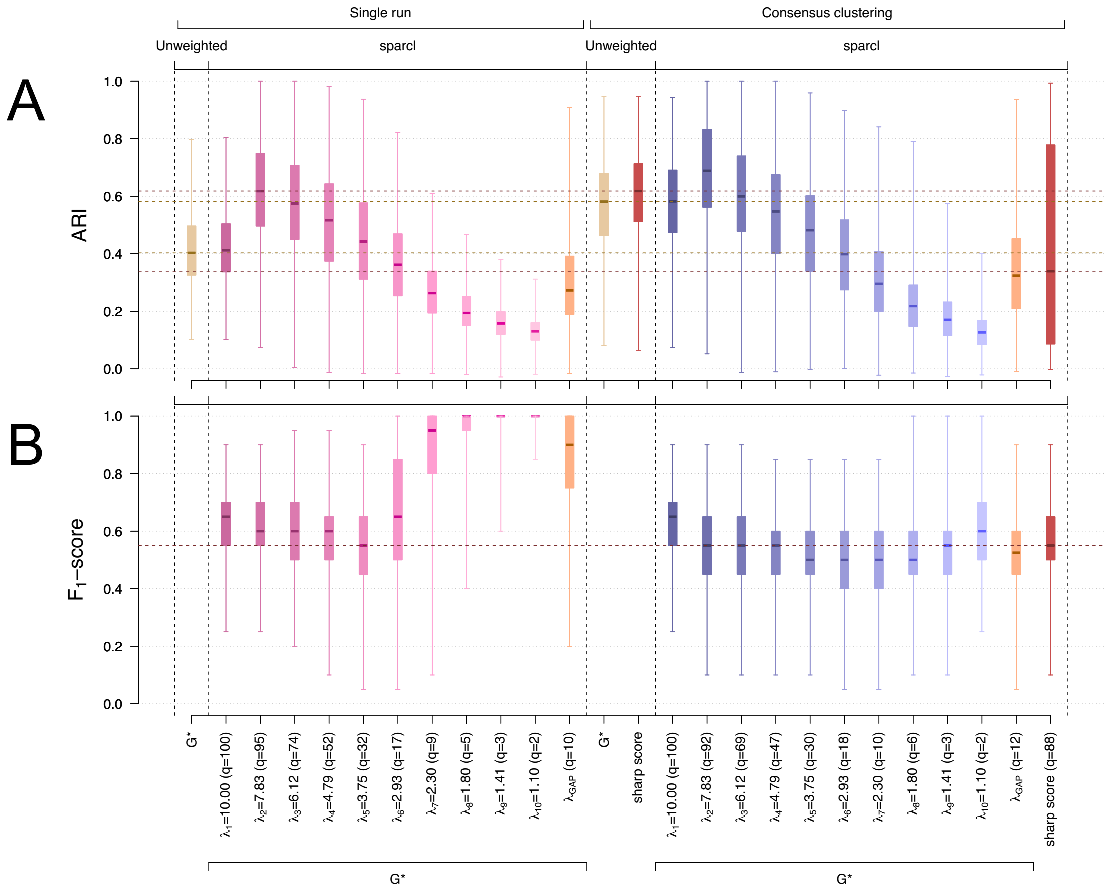

Supplementary Figure 12: Comparison of clustering performances by the Adjusted Rand Index (ARI) (A) and of the attribute weighting (when applicable) by the F_1-score (B) for (consensus) hierarchical clustering or sparse hierarchical. Performances are evaluated for $N = 1,000$ simulated datasets with $n = 150$ items split into $G^* = 5$ clusters including $N_1 = 20$, $N_2 = 50$, $N_3 = 30$, $N_4 = 10$, $N_5 = 40$ items, respectively. The clustering structure is supported by $q^* = 20$ of the $p = 100$ attributes with nonzero proportion of explained variance ($E = 0.5$). Hierarchical clustering is conducted using the true number of clusters G^* (in beige, pink or orange). For sparse clustering, we report performances using the true number of clusters G^* and (i) a range of regularisation parameters (in pink), or (ii) with the regularisation parameter calibrated by maximising the GAP statistic (reported as λ_{GAP}, in orange). For consensus unweighted clustering, we use the true number of clusters G^* (in beige) or calibrated number of clusters by maximising the sharp score (in red). For consensus weighted clustering, we (i) fix the number of clusters to G^* and consider ten different values of λ (in blue), (ii) use iteration-specific regularisation parameters calibrated by maximising the GAP statistic (λ_{GAP}, in orange), or (iii) jointly calibrate the number of clusters and the penalty parameter using our

sharp score (in red). For weighted clustering, the F_1-score measures weighting performance by comparing the sets of (i) the 20 features with highest selection proportions, and (ii) the 20 features supporting the clustering in the simulation (B).

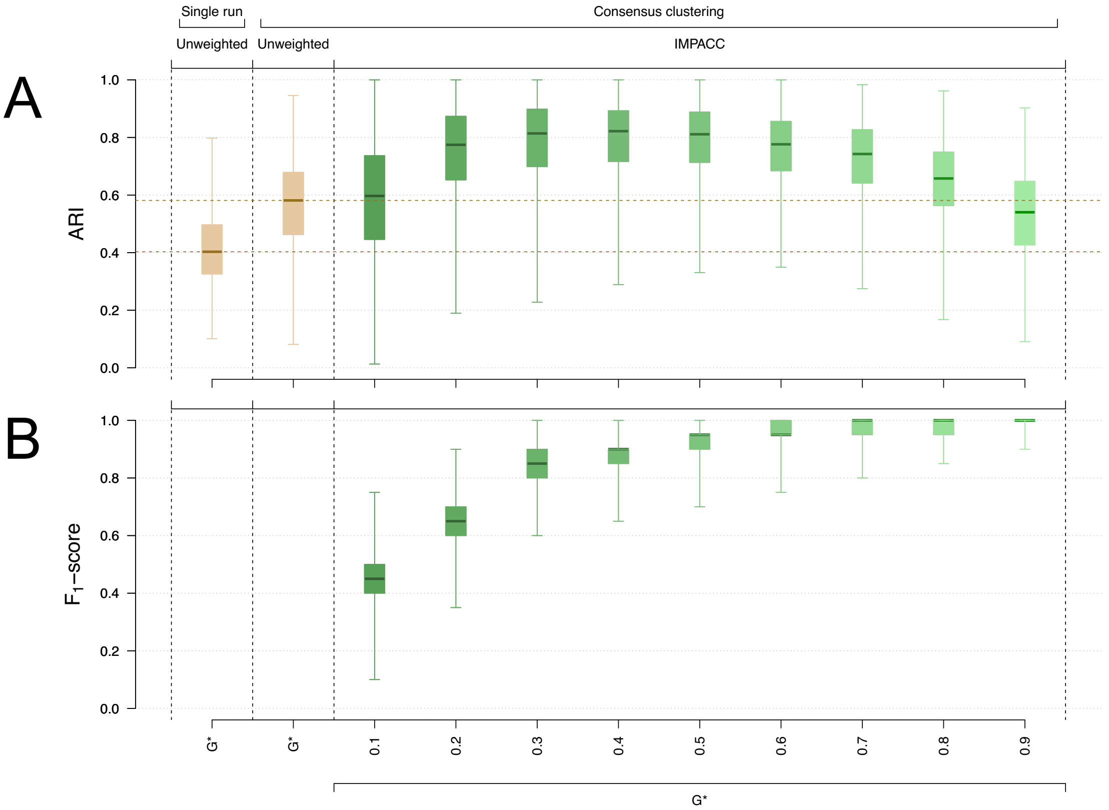

Supplementary Figure 13: Comparison of clustering performances by the Adjusted Rand Index (ARI) (A) and of the attribute weighting (when applicable) by the F_1-score (B) for hierarchical clustering, consensus hierarchical clustering or IMPACC. Performances are evaluated for $N = 1,000$ simulated datasets with $n = 150$ items split into $G^* = 5$ clusters including $N_1 = 20$, $N_2 = 50$, $N_3 = 30$, $N_4 = 10$, $N_5 = 40$ items, respectively. The clustering structure is supported by $q^* = 20$ of the $p = 100$ attributes with nonzero proportion of explained variance ($E = 0.5$). For (consensus) unweighted hierarchical clustering, we use the true number of clusters G^* (in beige) or calibrated number of clusters by maximising the sharp score (in red). For IMPACC, we fix the number of clusters to G^* and consider nine different proportions of attribute to sample (in green). For weighted clustering, the F_1-score measures weighting performance by comparing the sets of (i) the 20 features with highest selection proportions, and (ii) the 20 features supporting the clustering in the simulation (B).

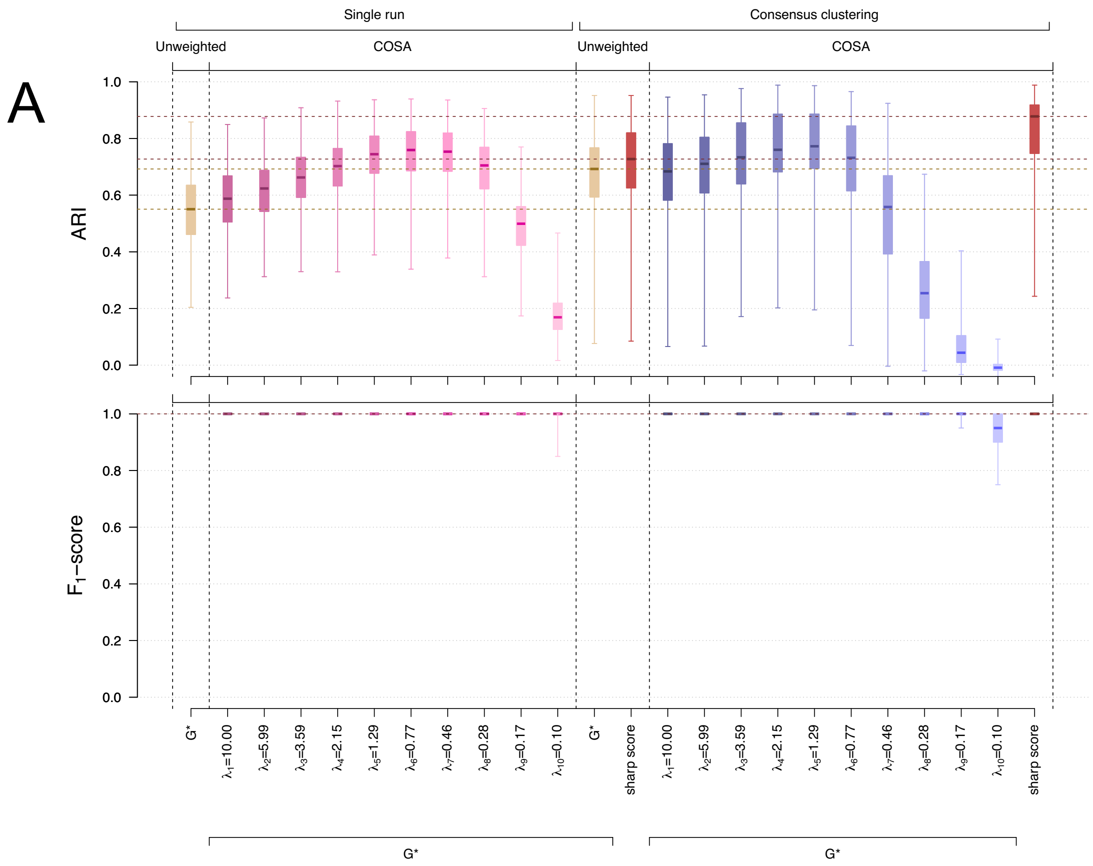
A
Single run
Consensus clustering
Unweighted
COSA
Unweighted
COSA
ARI
F$_1$−score
1.0
0.8
0.6
0.4
0.2
0.0
G*
λ$_1$=10.00
λ$_2$=5.99
λ$_3$=3.59
λ$_4$=2.15
λ$_5$=1.29
λ$_6$=0.77
λ$_7$=0.46
λ$_8$=0.28
λ$_9$=0.17
λ$_{10}$=0.10
sharp score
G*
G*

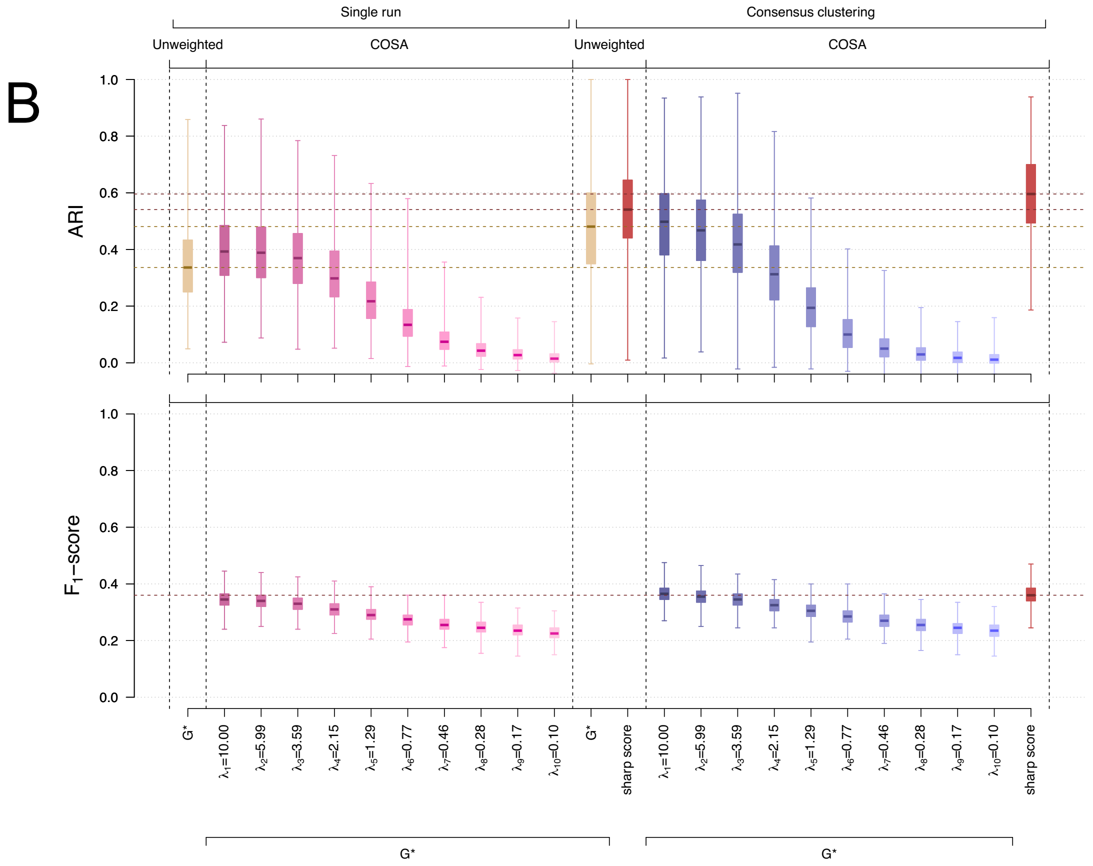
B
Single run
Consensus clustering
Unweighted
COSA
Unweighted
COSA
ARI
F1-score
1.0
0.8
0.6
0.4
0.2
0.0
G*
λ1=10.00
λ2=5.99
λ3=3.59
λ4=2.15
λ5=1.29
λ6=0.77
λ7=0.46
λ8=0.28
λ9=0.17
λ10=0.10
G*
sharp score
λ1=10.00
λ2=5.99
λ3=3.59
λ4=2.15
λ5=1.29
λ6=0.77
λ7=0.46
λ8=0.28
λ9=0.17
λ10=0.10
sharp score
G*
G*

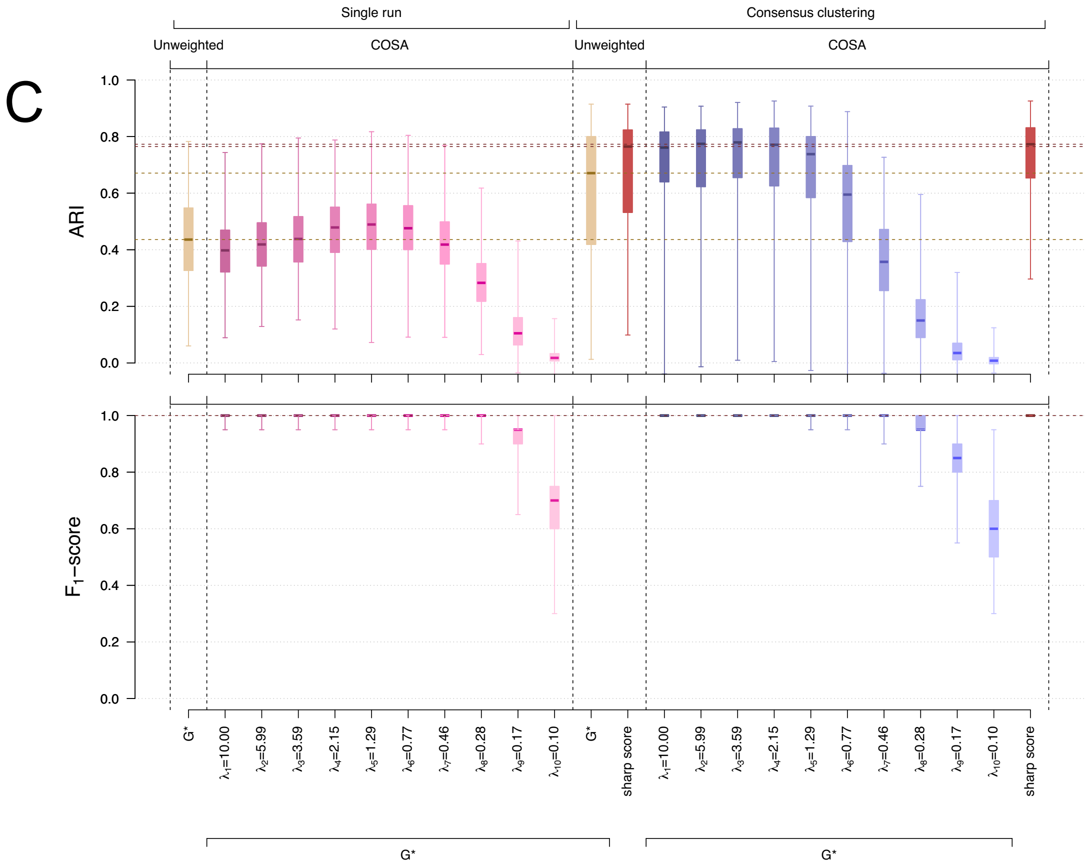

Supplementary Figure 14: Comparison of clustering performances by the Adjusted Rand Index (ARI) and of the attribute weighting (when applicable) by the F_1-score for hierarchical clustering using unweighted or COSA Euclidean distances and consensus hierarchical clustering using unweighted or COSA Euclidean distances. Performances are evaluated for $N = 1,000$ simulated datasets under three simulation scenarios. In a scenario with a large number of items (A), $n = 1,000$ items are split into $G^* = 5$ clusters including $N_1 = 133$, $N_2 = 333$, $N_3 = 200$, $N_4 = 67$, $N_5 = 267$ items, respectively, with a clustering structure supported by $q^* = 20$ of the $p = 100$ attributes with nonzero proportion of explained variance ($E = 0.5$). In a scenario with a large number of attributes (B), $n = 100$ items are split into $G^* = 5$ clusters including $N_1 = 13$, $N_2 = 33$, $N_3 = 20$, $N_4 = 7$, $N_5 = 27$ items, respectively, with a clustering structure supported by $q^* = 200$ of the $p = 1,000$ attributes with nonzero proportion of explained variance ($E = 0.15$). In a scenario with highly unbalanced clusters (C), $n = 1,000$ items are split into $G^* = 4$ clusters including $N_1 = 500$, $N_2 = 300$, $N_3 = 150$, and $N_4 = 50$ items, respectively, with a clustering structure supported by $q^* = 20$ of the $p = 100$ attributes with nonzero proportion of explained variance ($E = 0.35$). Hierarchical clustering is conducted using the true number of clusters G^*

(in beige or pink). For consensus unweighted clustering, we use the true number of clusters G^* (in beige) or calibrated number of clusters by maximising the sharp score (in red). For consensus weighted clustering, we (i) fix the number of clusters to G^* and consider ten different values of λ (in blue), or (ii) jointly calibrate the number of clusters and the penalty parameter using our sharp score (in red). For weighted clustering, the F_1-score measures weighting performance by comparing the sets of (i) the q^* features with highest weights, and (ii) the q^* features supporting the clustering in the simulation (B).

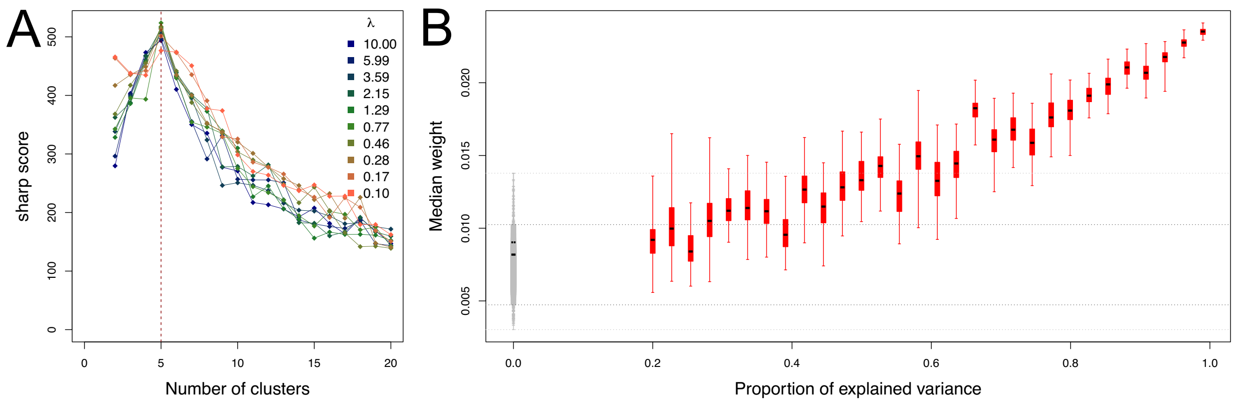

Supplementary Figure 15: Distribution of median weights obtained from consensus COSA clustering as a function of the simulated proportion of explained variance by feature. The simulated datasets has $n = 150$ items split into $G^* = 5$ such that $N_1 = 20$, $N_2 = 50$, $N_3 = 30$, $N_4 = 10$, $N_5 = 40$ across $p = 100$ features, of which $q^* = 30$ had a nonzero proportion of explained variance (ranging from $E = 0.2$ to $E = 0.99$). Calibration curves show the sharp score as a function of the number of clusters (A). Boxes showing the distribution of (median) feature weights are coloured in red for contributing features ($E \neq 0$) and grey for non-contributing features ($E = 0$) (B). Features are ordered by proportion of simulated explained variance.

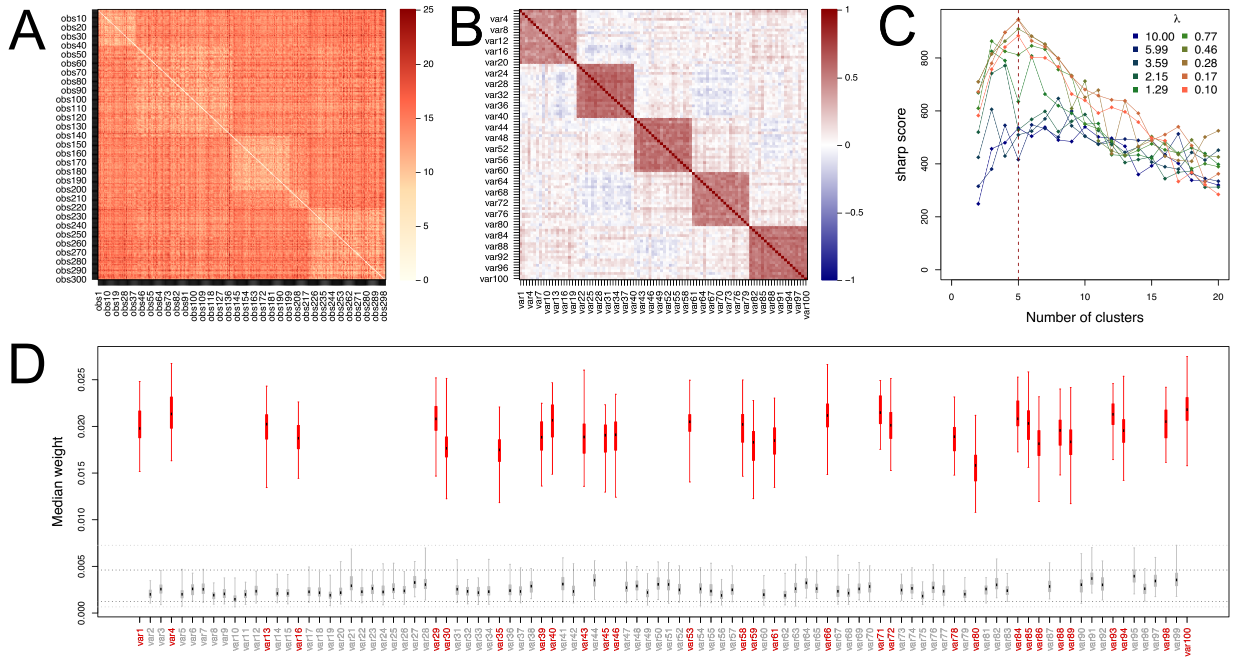

Supplementary Figure 16: Distribution of median weights obtained from consensus COSA clustering on simulated data with correlated features. The simulated datasets has $n = 300$ items split into $G^* = 5$ such that $N_1 = 40$, $N_2 = 100$, $N_3 = 60$, $N_4 = 20$, $N_5 = 80$ across $p = 100$ features, of which $q^* = 30$ had a nonzero proportion of explained variance ($E = 0.7$). We show heatmaps of Euclidean distances between items (A) and Pearson's correlations between features (B). Calibration curves show the sharp score as a function of the number of clusters (C). Boxes showing the distribution of (median) feature weights are coloured in red for contributing features ($E = 0.7$) and grey for non-contributing features ($E = 0$) (D).

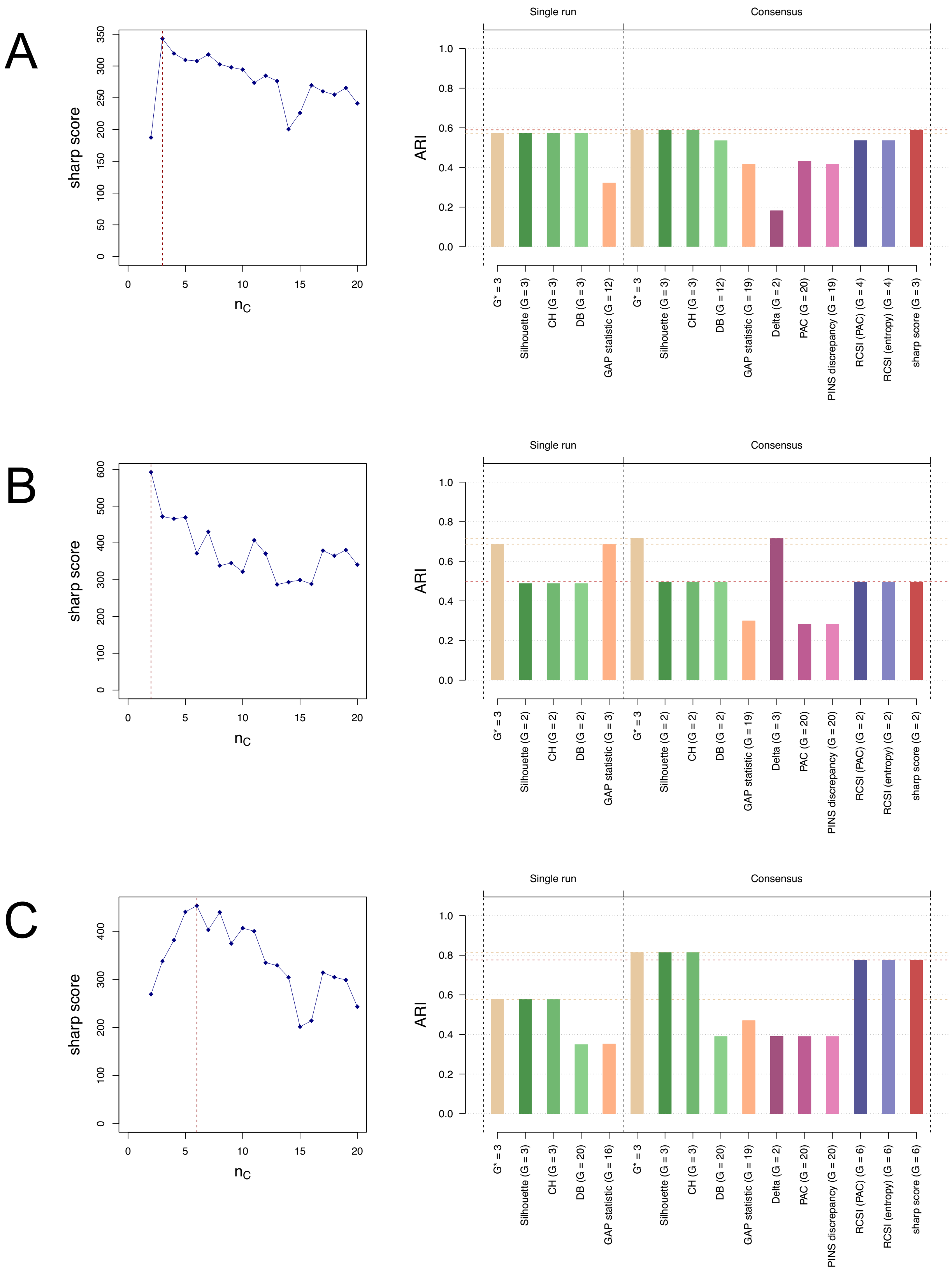
A
B
C
sharp score
n_C
ARI
Single run
Consensus
A: G* = 3, Silhouette (G = 3), CH (G = 3), DB (G = 3), GAP statistic (G = 12), G* = 3, Silhouette (G = 3), CH (G = 3), DB (G = 12), GAP statistic (G = 19), Delta (G = 2), PAC (G = 20), PINS discrepancy (G = 19), RCSI (PAC) (G = 4), RCSI (entropy) (G = 4), sharp score (G = 3)
B: G* = 3, Silhouette (G = 2), CH (G = 2), DB (G = 2), GAP statistic (G = 3), G* = 3, Silhouette (G = 2), CH (G = 2), DB (G = 2), GAP statistic (G = 19), Delta (G = 3), PAC (G = 20), PINS discrepancy (G = 20), RCSI (PAC) (G = 2), RCSI (entropy) (G = 2), sharp score (G = 2)
C: G* = 3, Silhouette (G = 3), CH (G = 3), DB (G = 20), GAP statistic (G = 16), G* = 3, Silhouette (G = 3), CH (G = 3), DB (G = 20), GAP statistic (G = 19), Delta (G = 2), PAC (G = 20), PINS discrepancy (G = 20), RCSI (PAC) (G = 6), RCSI (entropy) (G = 6), sharp score (G = 6)

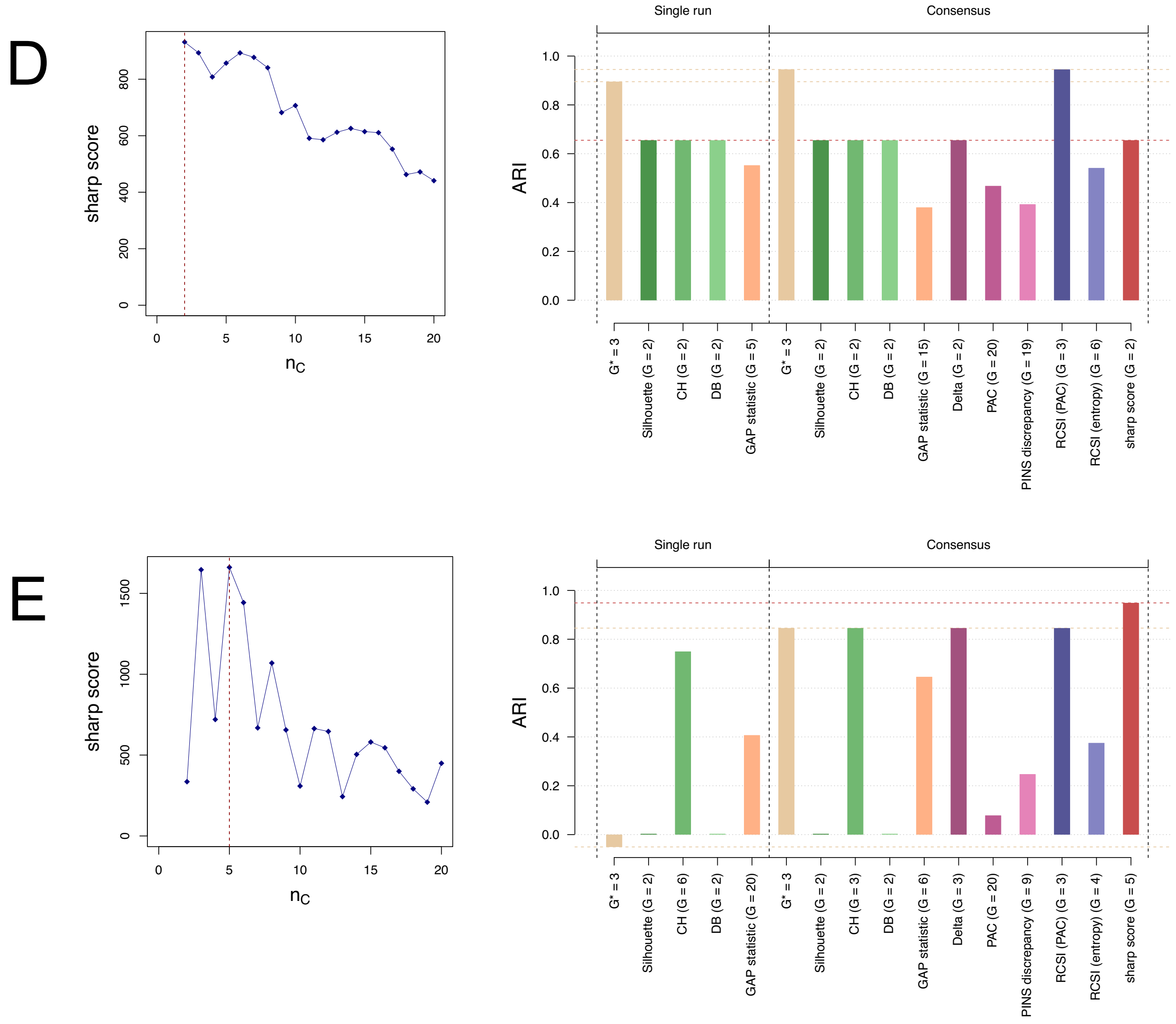

Supplementary Figure 17: Calibration curve showing the sharp score as a function of the number of clusters (left) and Adjusted Rand Index (ARI) obtained with different unweighted clustering approaches (right) on five publicly available real datasets. We use (consensus) hierarchical clustering on the iris (A), seeds (B), wine (C), palmer penguins (D), and hawks (E) datasets. The ARI is calculated by comparing the clusters and true classes of items for hierarchical clustering with the simulated number of clusters (G^*), or calibrated by maximising the silhouette, Calinski–Harabasz (CH), Davies–Bouldin (DB) and GAP score, and for consensus hierarchical clustering with G^* or calibrated using the silhouette, Calinski–Harabasz (CH), Davies–Bouldin (DB), Δ, PAC, PINS discrepancy, RCSI and sharp scores. The calibrated number of clusters using each method is reported in brackets.

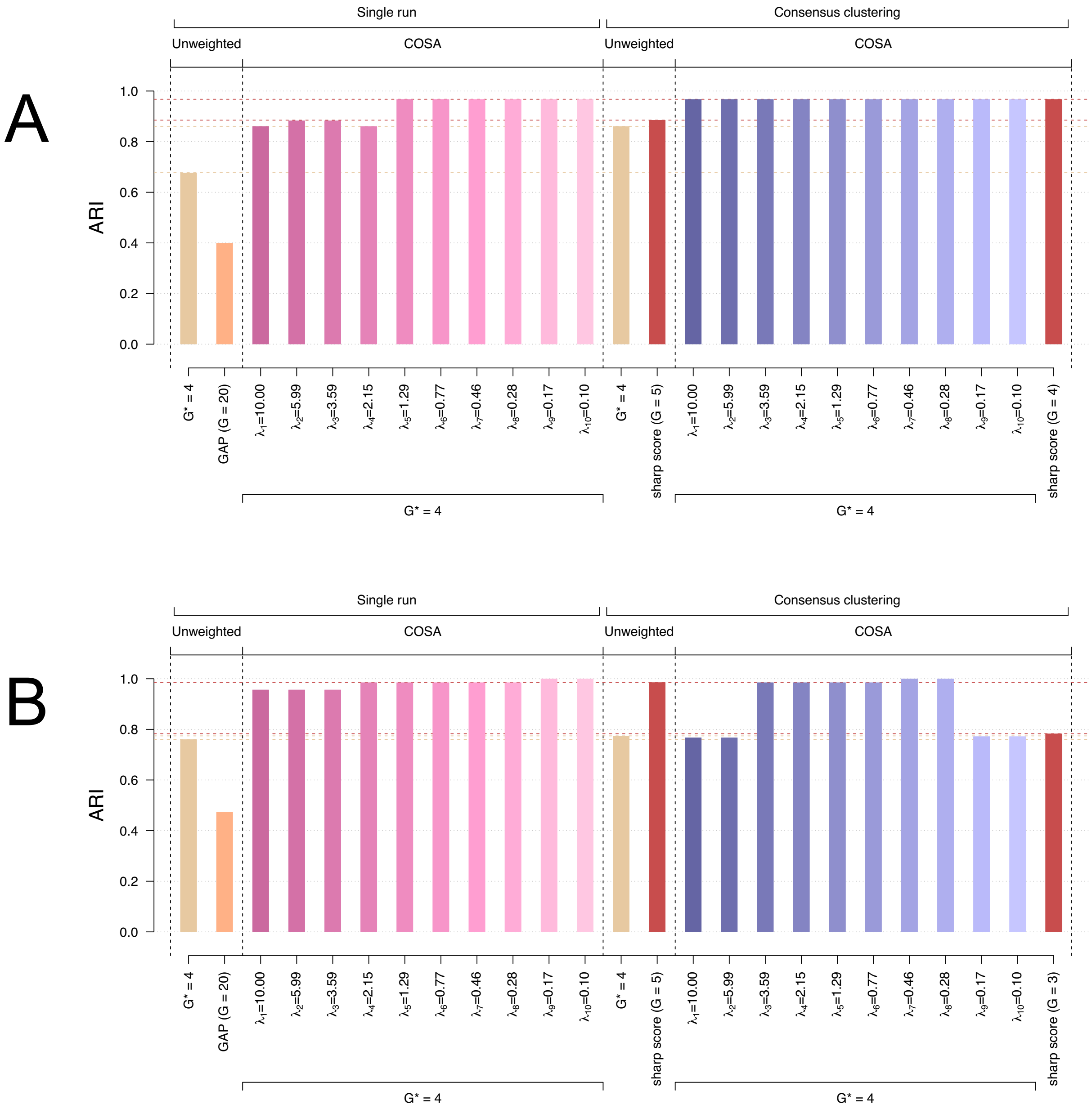
A
B
Single run
Consensus clustering
Unweighted
COSA
ARI
G* = 4
GAP (G = 20)
sharp score (G = 5)
sharp score (G = 4)
sharp score (G = 3)
λ1=10.00
λ2=5.99
λ3=3.59
λ4=2.15
λ5=1.29
λ6=0.77
λ7=0.46
λ8=0.28
λ9=0.17
λ10=0.10

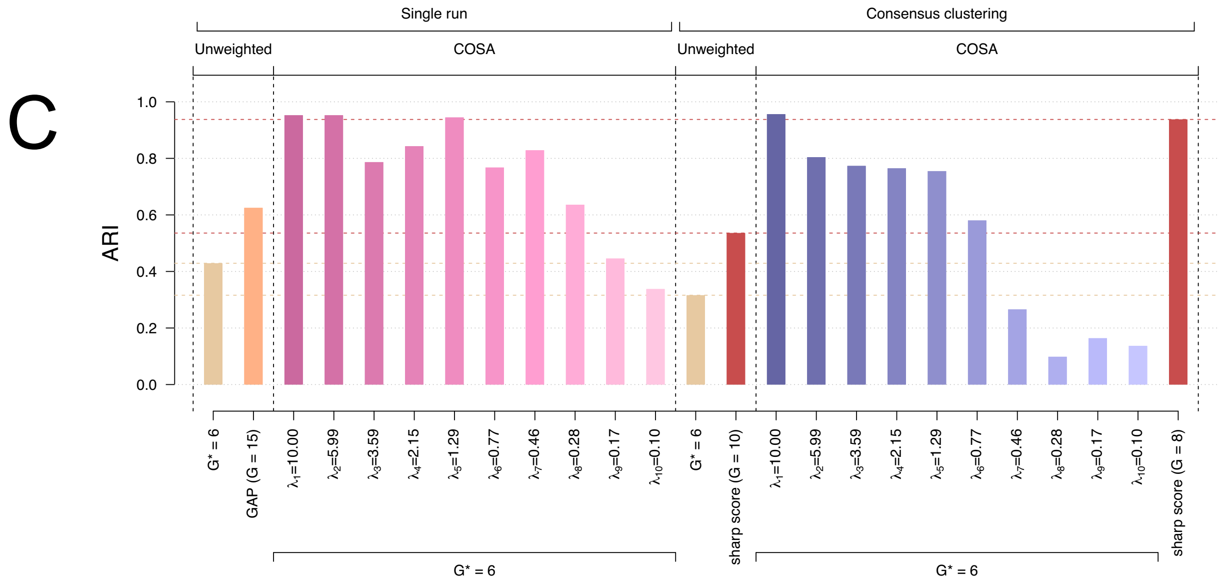

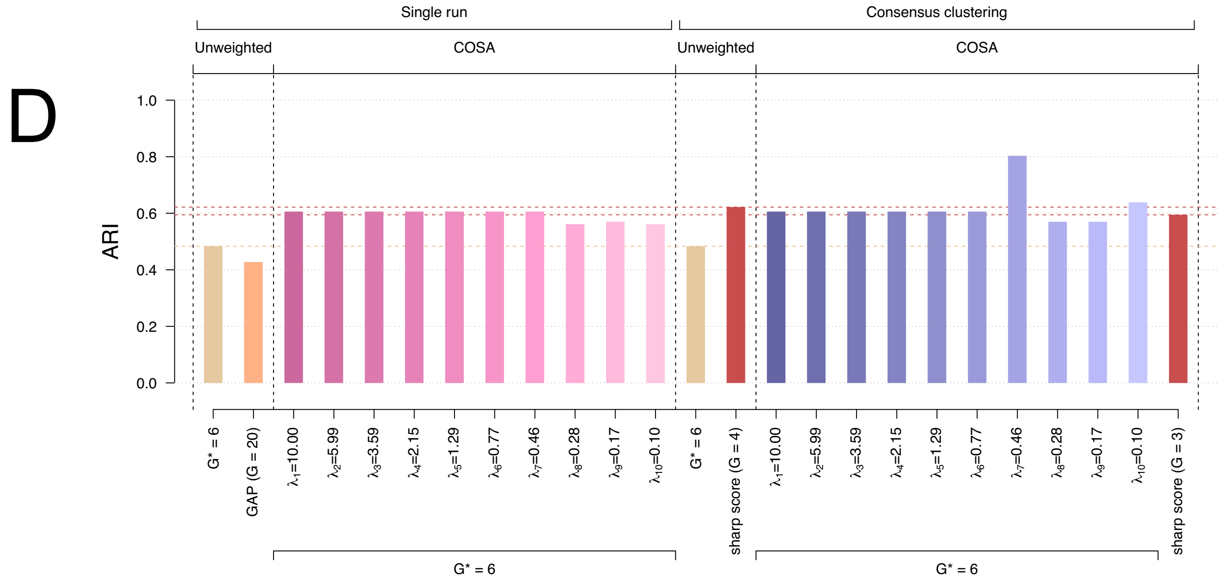

Supplementary Figure 18: Adjusted Rand Index (ARI) obtained with different (weighted) hierarchical clustering approaches on four publicly available molecular datasets. We use (consensus COSA) hierarchical clustering on microarray data from lung tissue samples (A), bulk RNAseq data from tumours of different cancer types (B), the Tabula Muris single cell RNAseq data with multiple cell lines (C), and the Human single cell RNAseq data with different cell types (D). The ARI is calculated by comparing the clusters and true classes of items for hierarchical clustering with the simulated number of clusters (G^*, in beige), or calibrated by maximising the GAP score (in orange), for hierarchical COSA clustering using G^* and different values of the regularisation parameter λ (in pink), for consensus hierarchical clustering with G^*

(in beige) or calibrated using the sharp score (in red), and for consensus hierarchical COSA clustering using G^* and different values of λ (in blue), or calibrated using the sharp score (in red). The calibrated number of clusters using each method is reported in brackets.

A

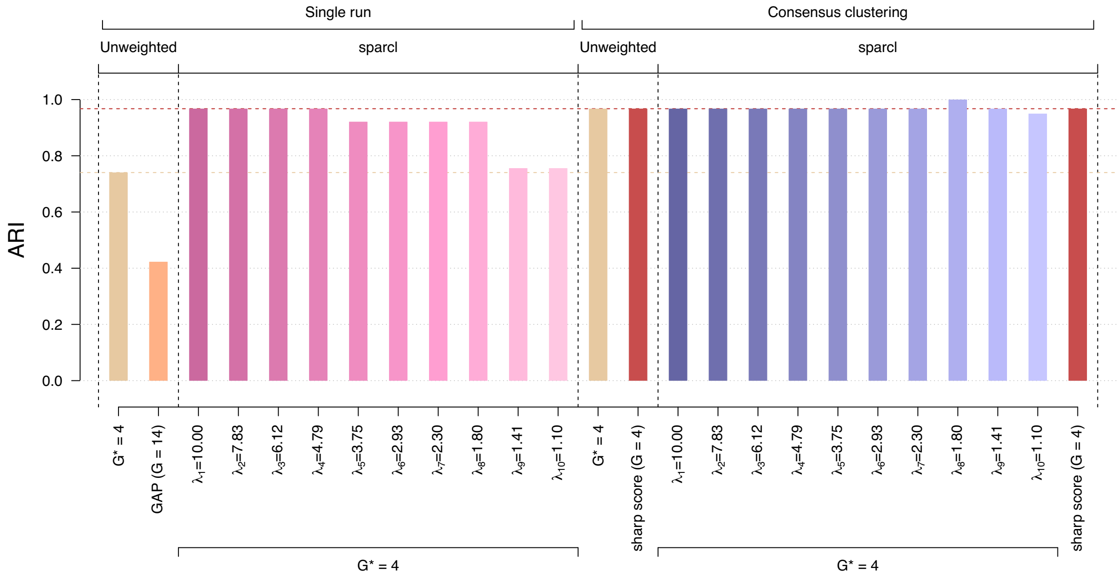

Single run
Consensus clustering
Unweighted
sparcl
Unweighted
sparcl
ARI
1.0
0.8
0.6
0.4
0.2
0.0
G* = 4
GAP (G = 14)
λ1=10.00
λ2=7.83
λ3=6.12
λ4=4.79
λ5=3.75
λ6=2.93
λ7=2.30
λ8=1.80
λ9=1.41
λ10=1.10
G* = 4
sharp score (G = 4)
λ1=10.00
λ2=7.83
λ3=6.12
λ4=4.79
λ5=3.75
λ6=2.93
λ7=2.30
λ8=1.80
λ9=1.41
λ10=1.10
sharp score (G = 4)
G* = 4
G* = 4

B

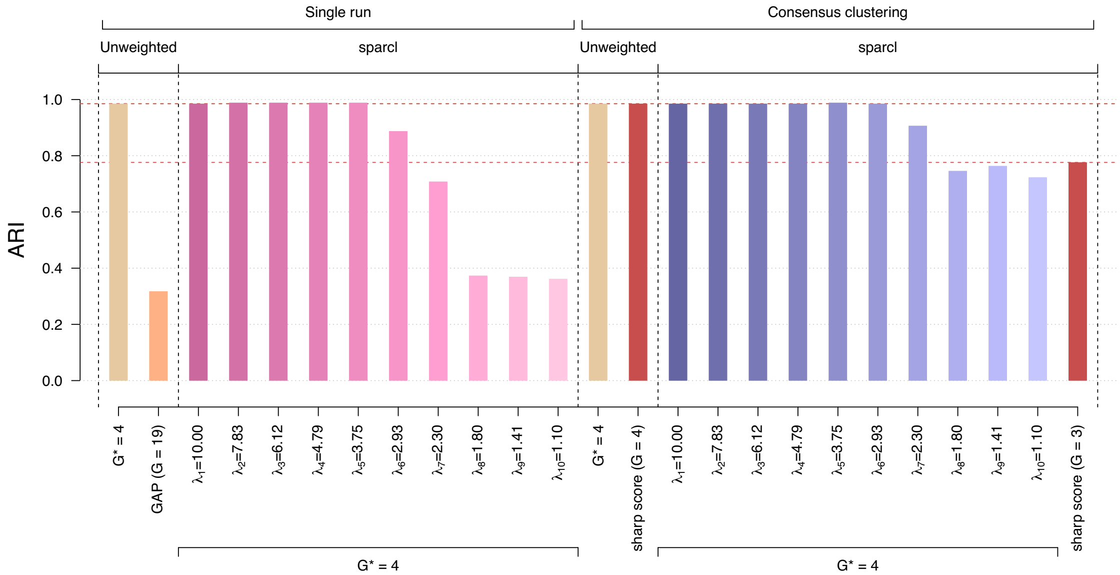

Single run
Consensus clustering
Unweighted
sparcl
Unweighted
sparcl
ARI
1.0
0.8
0.6
0.4
0.2
0.0
G* = 4
GAP (G = 19)
λ1=10.00
λ2=7.83
λ3=6.12
λ4=4.79
λ5=3.75
λ6=2.93
λ7=2.30
λ8=1.80
λ9=1.41
λ10=1.10
G* = 4
sharp score (G = 4)
λ1=10.00
λ2=7.83
λ3=6.12
λ4=4.79
λ5=3.75
λ6=2.93
λ7=2.30
λ8=1.80
λ9=1.41
λ10=1.10
sharp score (G = 3)
G* = 4
G* = 4

C

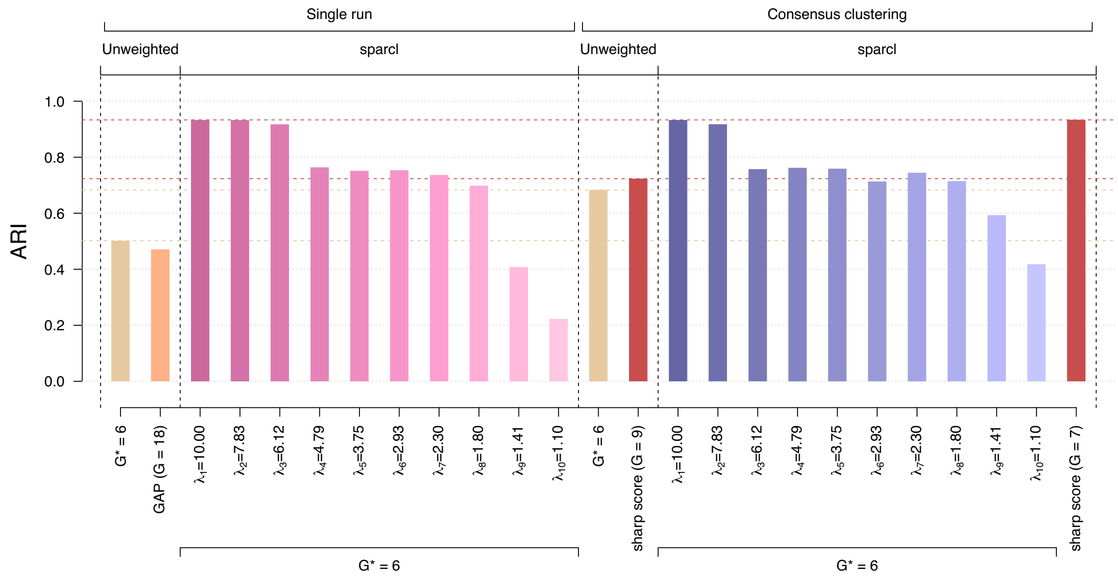

D

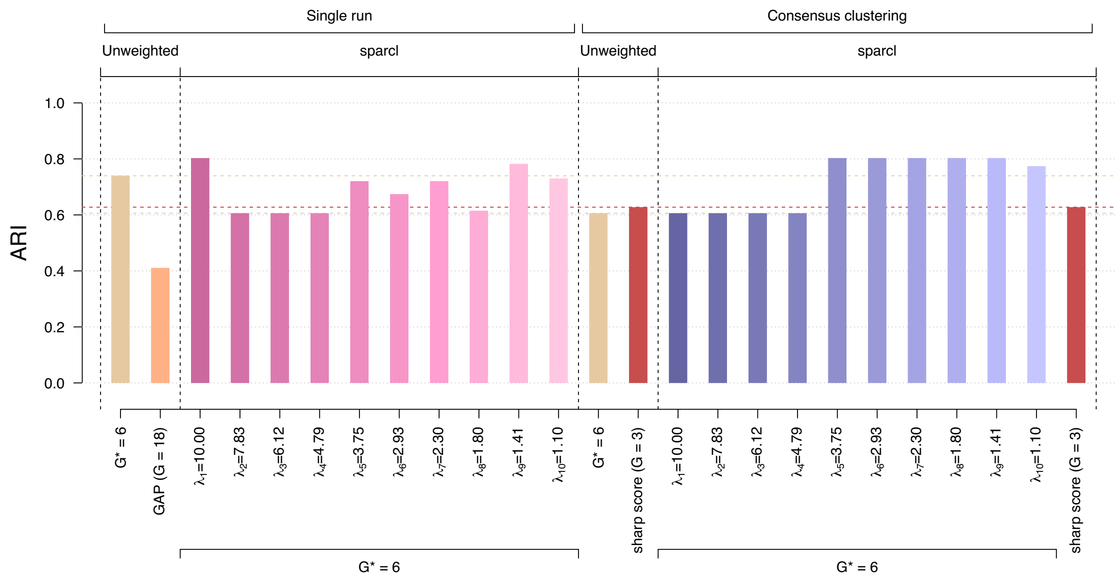

Supplementary Figure 19: Adjusted Rand Index (ARI) obtained with different (sparse) K means approaches on four publicly available molecular datasets. We use (consensus sparse) K means on microarray data from lung tissue samples (A), bulk RNAseq data from tumours of different cancer types (B), the Tabula Muris single cell RNAseq data with multiple cell lines (C), and the Human single cell RNAseq data with different cell types (D). The ARI is calculated by comparing the clusters and true classes of items for K means with the simulated number of clusters (G^*, in beige), or calibrated by maximising the GAP score (in orange), for sparse K means using G^* and different values of the regularisation parameter λ (in pink), for consensus K means with G^* (in beige) or calibrated using the sharp score (in red), and for consensus

sparse K means using G^* and different values of λ (in blue), or calibrated using the sharp score (in red). The calibrated number of clusters using each method is reported in brackets.

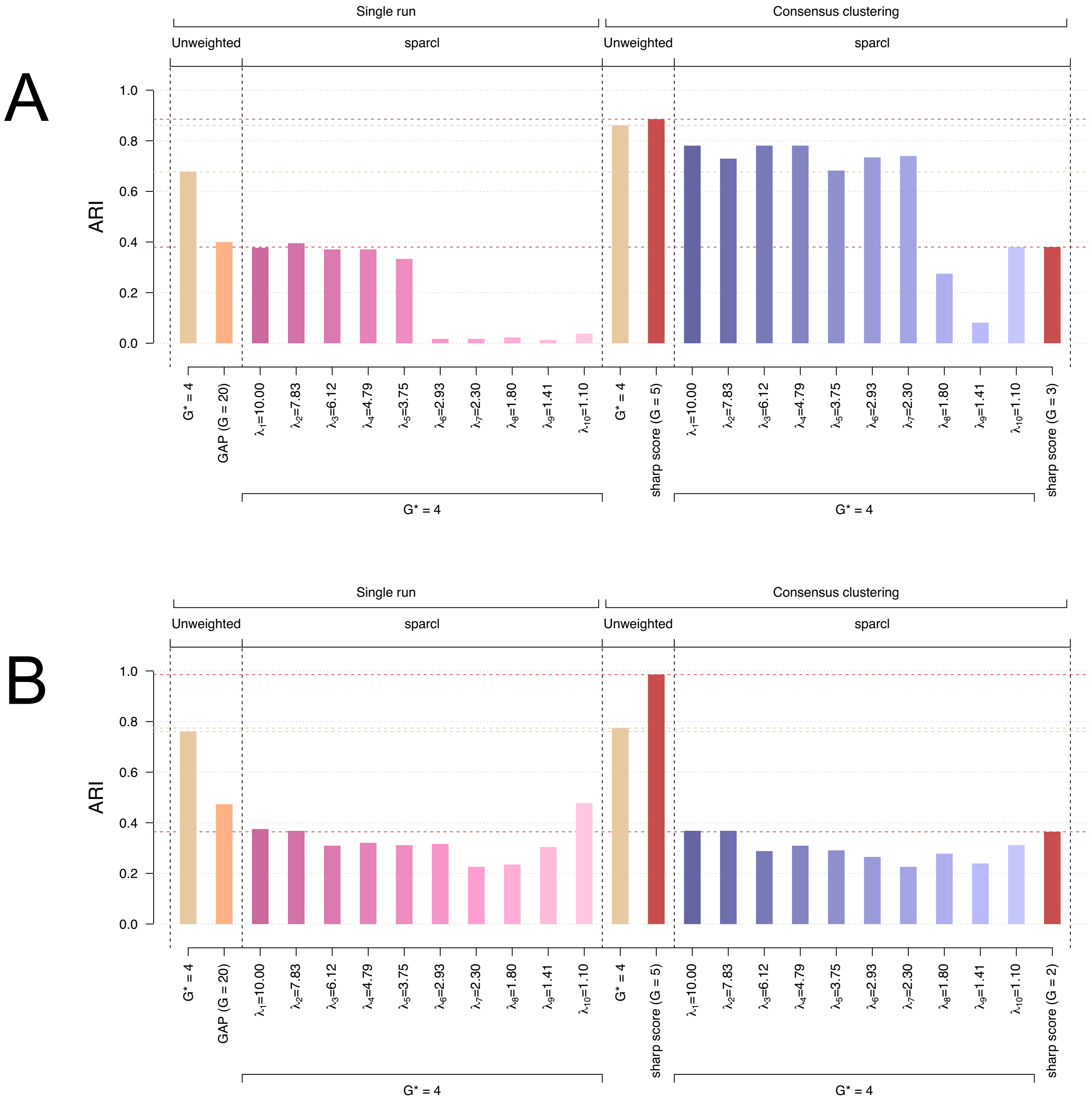

A
Single run
Consensus clustering
Unweighted
sparcl
Unweighted
sparcl
ARI
1.0
0.8
0.6
0.4
0.2
0.0
G* = 4
GAP (G = 20)
λ_1=10.00
λ_2=7.83
λ_3=6.12
λ_4=4.79
λ_5=3.75
λ_6=2.93
λ_7=2.30
λ_8=1.80
λ_9=1.41
λ_{10}=1.10
G* = 4
sharp score (G = 5)
λ_1=10.00
λ_2=7.83
λ_3=6.12
λ_4=4.79
λ_5=3.75
λ_6=2.93
λ_7=2.30
λ_8=1.80
λ_9=1.41
λ_{10}=1.10
sharp score (G = 3)
G* = 4
G* = 4
B
Single run
Consensus clustering
Unweighted
sparcl
Unweighted
sparcl
ARI
1.0
0.8
0.6
0.4
0.2
0.0
G* = 4
GAP (G = 20)
λ_1=10.00
λ_2=7.83
λ_3=6.12
λ_4=4.79
λ_5=3.75
λ_6=2.93
λ_7=2.30
λ_8=1.80
λ_9=1.41
λ_{10}=1.10
G* = 4
sharp score (G = 5)
λ_1=10.00
λ_2=7.83
λ_3=6.12
λ_4=4.79
λ_5=3.75
λ_6=2.93
λ_7=2.30
λ_8=1.80
λ_9=1.41
λ_{10}=1.10
sharp score (G = 2)
G* = 4
G* = 4

C

D

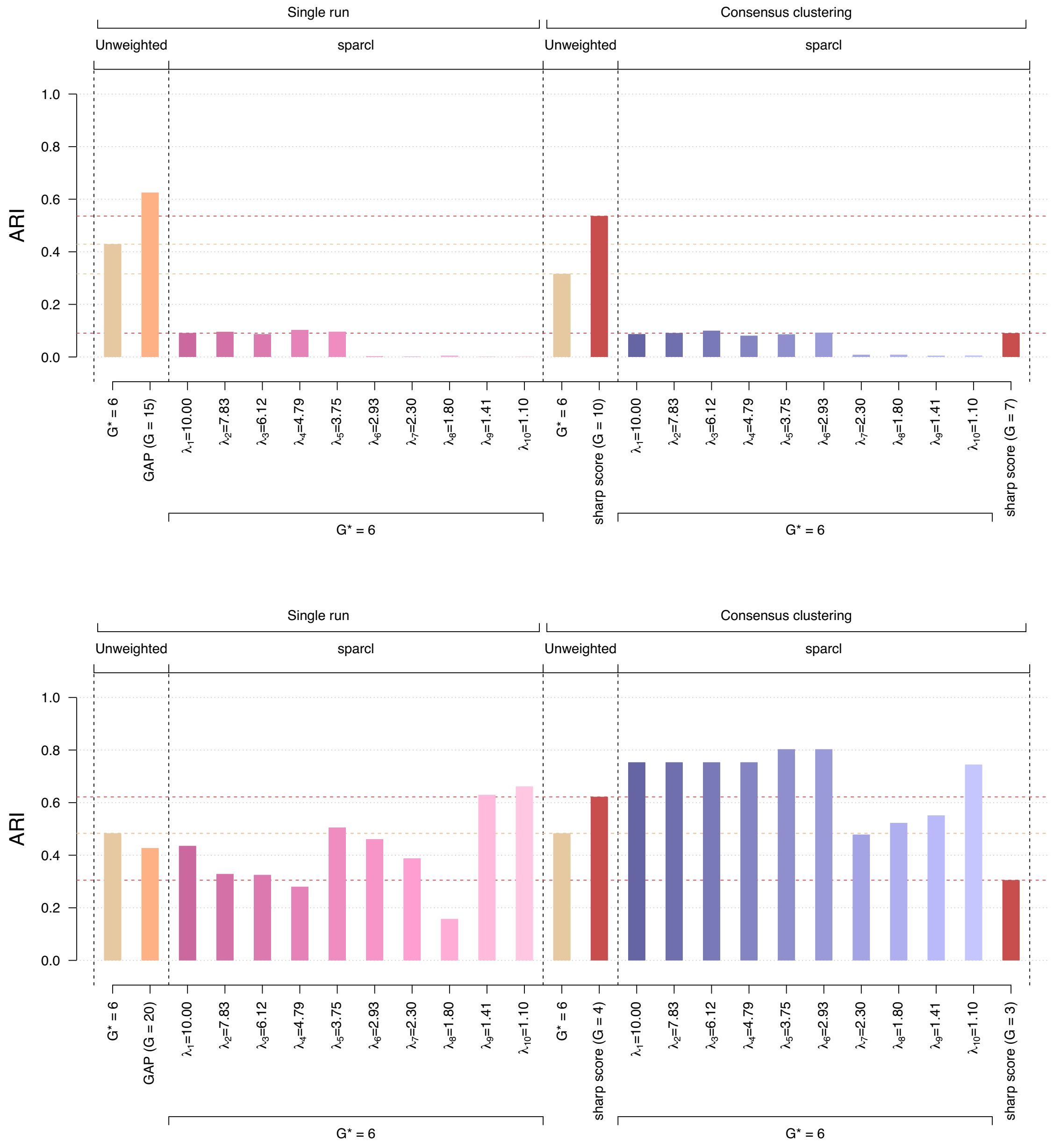

Supplementary Figure 20: Adjusted Rand Index (ARI) obtained with different (sparse) hierarchical clustering approaches on four publicly available molecular datasets. We use (consensus sparse) hierarchical clustering on microarray data from lung tissue samples (A), bulk RNAseq data from tumours of different cancer types (B), the Tabula Muris single cell RNAseq data with multiple cell lines (C), and the Human single cell RNAseq data with different cell types (D). The ARI is calculated by comparing the clusters and true classes of items for hierarchical clustering with the simulated number of clusters (G^*, in beige), or calibrated by maximising the GAP score (in orange), for sparse hierarchical clustering using G^* and different values of the regularisation parameter λ (in pink), for consensus hierarchical clustering with

G^* (in beige) or calibrated using the sharp score (in red), and for consensus sparse hierarchical clustering using G^* and different values of λ (in blue), or calibrated using the sharp score (in red). The calibrated number of clusters using each method is reported in brackets.

A

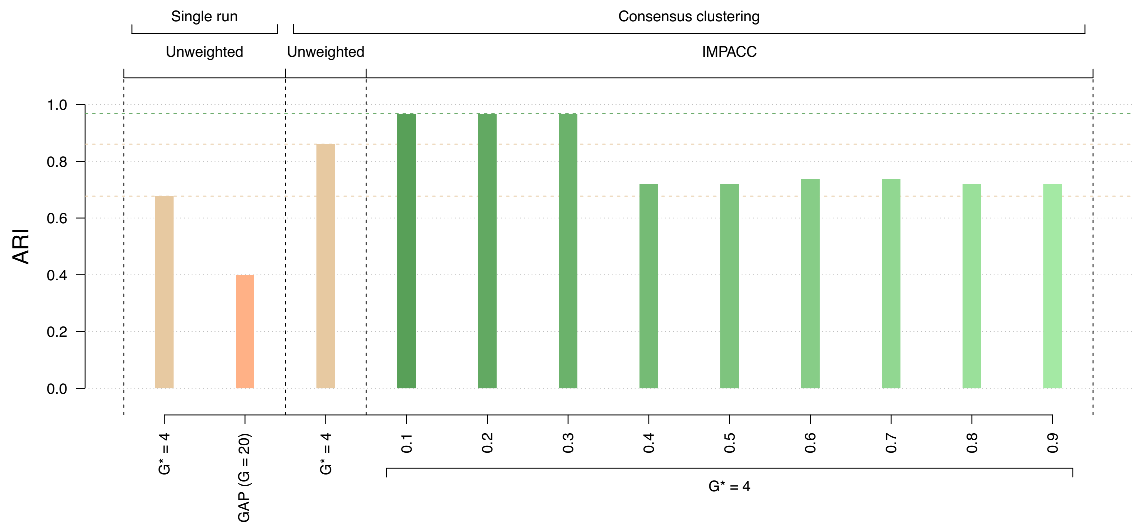
Single run
Consensus clustering
Unweighted
Unweighted
IMPACC
ARI
1.0
0.8
0.6
0.4
0.2
0.0
G* = 4
GAP (G = 20)
G* = 4
0.1
0.2
0.3
0.4
0.5
0.6
0.7
0.8
0.9
G* = 4

B

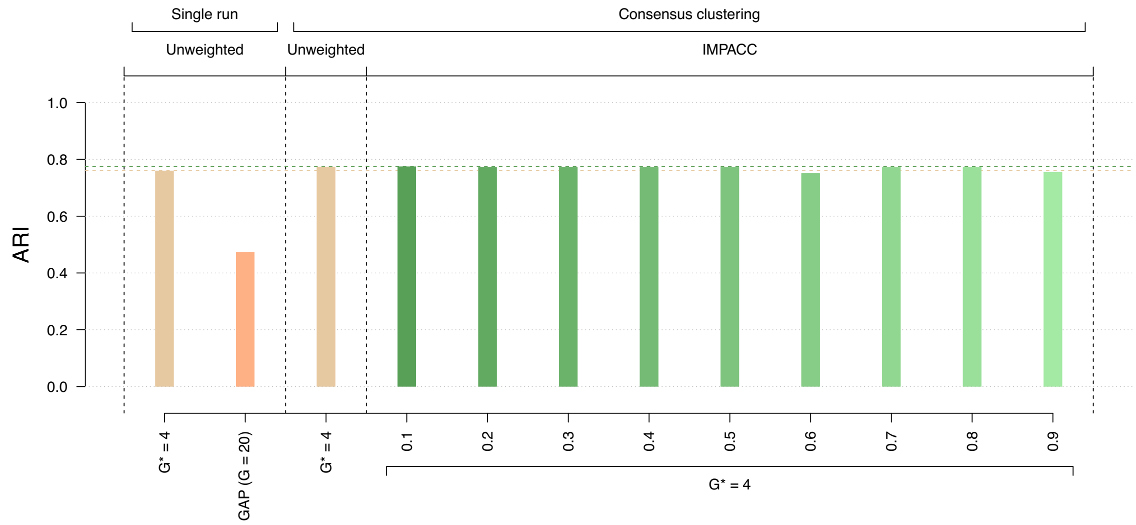
Single run
Consensus clustering
Unweighted
Unweighted
IMPACC
ARI
1.0
0.8
0.6
0.4
0.2
0.0
G* = 4
GAP (G = 20)
G* = 4
0.1
0.2
0.3
0.4
0.5
0.6
0.7
0.8
0.9
G* = 4

C

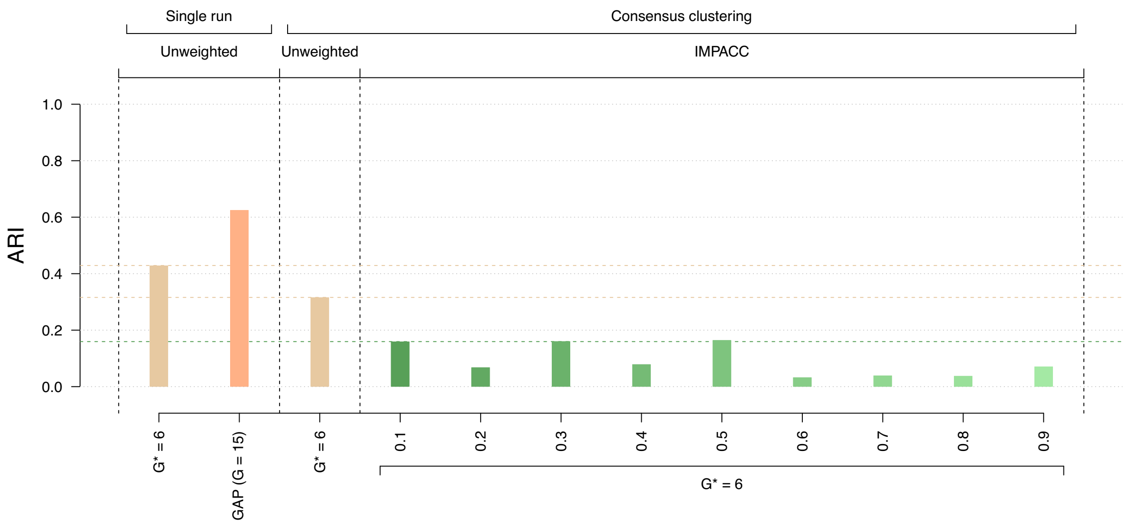

D

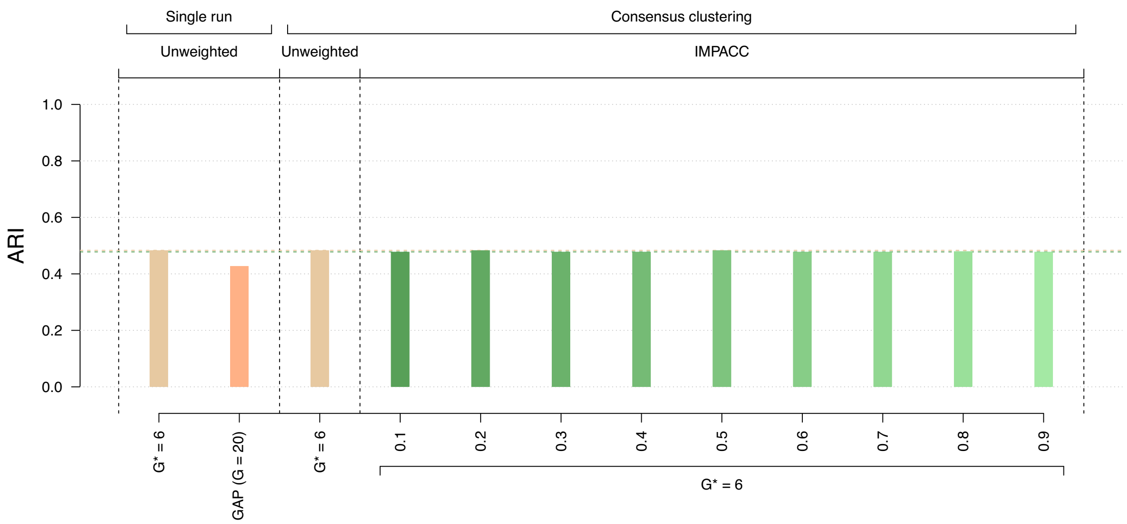

Supplementary Figure 21: Adjusted Rand Index (ARI) obtained with different (weighted) hierarchical clustering approaches on four publicly available molecular datasets. We use (consensus) hierarchical clustering and IMPACC on microarray data from lung tissue samples (A), bulk RNAseq data from tumours of different cancer types (B), the Tabula Muris single cell RNAseq data with multiple cell lines (C), and the Human single cell RNAseq data with different cell types (D). The ARI is calculated by comparing the clusters and true classes of items for (consensus) hierarchical clustering with the simulated number of clusters (G^*, in beige). For IMPACC, we use G^* and different values of the proportion of attributes to sample (in green). The calibrated number of clusters using each method is reported in brackets.

3 Supplementary tables

E	Method	G	Rand index	ARI	Jaccard index	Percentage	Time (s)
	G*	5 [0]	0.971 [0.029]	0.921 [0.079]	0.887 [0.106]		0 [0]
	Silhouette	5 [1]	0.957 [0.110]	0.884 [0.251]	0.840 [0.275]		0 [0]
	CH	4 [2]	0.943 [0.100]	0.848 [0.227]	0.794 [0.249]		0 [0]
	DB	4 [2]	0.943 [0.200]	0.851 [0.409]	0.798 [0.399]		0 [0]
	GAP statistic	5 [0]	0.972 [0.027]	0.922 [0.075]	0.887 [0.100]		2 [1]
	G*	5 [0]	0.984 [0.018]	0.957 [0.050]	0.936 [0.071]		5 [4]
	Silhouette	5 [1]	0.973 [0.094]	0.926 [0.224]	0.894 [0.263]		5 [4]
0.6	CH	4 [2]	0.957 [0.101]	0.887 [0.239]	0.844 [0.271]		5 [4]
	DB	5 [2]	0.967 [0.167]	0.912 [0.358]	0.875 [0.378]		5 [4]
	GAP statistic	11 [4]	0.880 [0.060]	0.606 [0.235]	0.506 [0.243]		5 [4]
	Delta	3 [1]	0.740 [0.127]	0.480 [0.199]	0.480 [0.139]		5 [4]
	PAC	5 [0]	0.978 [0.192]	0.940 [0.493]	0.912 [0.481]		5 [4]
	PINS discrepancy	5 [1]	0.978 [0.107]	0.939 [0.247]	0.911 [0.289]		5 [4]
	RCSI (PAC)	5 [1]	0.979 [0.049]	0.942 [0.126]	0.916 [0.168]	100%	378 [306]
	RCSI (entropy)	5 [1]	0.980 [0.038]	0.945 [0.100]	0.919 [0.135]	100%	426 [278]
	sharp score	5 [1]	0.979 [0.053]	0.943 [0.134]	0.917 [0.173]		5 [4]
	G*	5 [0]	0.916 [0.053]	0.770 [0.143]	0.703 [0.156]		0 [0]
	Silhouette	4 [3]	0.866 [0.237]	0.664 [0.403]	0.607 [0.314]		0 [0]
	CH	3 [2]	0.840 [0.197]	0.617 [0.327]	0.565 [0.248]		0 [0]
	DB	4 [2]	0.858 [0.252]	0.656 [0.440]	0.600 [0.342]		0 [0]
	GAP statistic	5 [0]	0.918 [0.047]	0.776 [0.132]	0.707 [0.147]		1 [0]
	G*	5 [0]	0.948 [0.037]	0.857 [0.100]	0.805 [0.122]		4 [2]
	Silhouette	4 [2]	0.916 [0.191]	0.778 [0.370]	0.717 [0.336]		4 [2]
0.5	CH	3 [2]	0.862 [0.216]	0.670 [0.386]	0.618 [0.320]		4 [2]
	DB	4 [2]	0.892 [0.261]	0.733 [0.537]	0.675 [0.459]		4 [2]
	GAP statistic	13 [7]	0.836 [0.078]	0.446 [0.277]	0.360 [0.234]		4 [2]
	Delta	3 [1]	0.730 [0.123]	0.456 [0.176]	0.463 [0.116]		4 [2]
	PAC	20 [0]	0.770 [0.032]	0.280 [0.102]	0.267 [0.081]		4 [2]
	PINS discrepancy	20 [0]	0.771 [0.034]	0.283 [0.114]	0.274 [0.092]		4 [2]
	RCSI (PAC)	5 [1]	0.936 [0.083]	0.826 [0.200]	0.767 [0.218]	100%	219 [209]
	RCSI (entropy)	5 [1]	0.939 [0.080]	0.832 [0.195]	0.774 [0.212]	100%	237 [248]
	sharp score	5 [1]	0.940 [0.085]	0.836 [0.200]	0.779 [0.216]		4 [2]
	G*	5 [0]	0.831 [0.060]	0.540 [0.152]	0.485 [0.126]		0 [0]
	Silhouette	3 [2]	0.712 [0.262]	0.391 [0.337]	0.408 [0.186]		0 [0]
	CH	3 [2]	0.720 [0.177]	0.391 [0.236]	0.407 [0.133]		0 [0]
	DB	7 [17]	0.794 [0.073]	0.281 [0.257]	0.274 [0.252]		0 [0]
	GAP statistic	5 [2]	0.840 [0.050]	0.555 [0.144]	0.497 [0.130]		2 [1]
	G*	5 [0]	0.874 [0.064]	0.655 [0.164]	0.586 [0.152]		5 [3]
	Silhouette	3 [2]	0.784 [0.264]	0.503 [0.391]	0.481 [0.255]		5 [3]
0.4	CH	3 [2]	0.751 [0.176]	0.451 [0.261]	0.450 [0.167]		5 [3]
	DB	17 [16]	0.731 [0.093]	0.235 [0.212]	0.277 [0.143]		5 [3]
	GAP statistic	17 [6]	0.772 [0.053]	0.255 [0.152]	0.235 [0.101]		5 [3]
	Delta	3 [1]	0.707 [0.117]	0.405 [0.163]	0.423 [0.100]		5 [3]
	PAC	20 [0]	0.736 [0.038]	0.197 [0.090]	0.218 [0.065]		5 [3]
	PINS discrepancy	20 [0]	0.736 [0.039]	0.197 [0.091]	0.218 [0.067]		5 [3]
	RCSI (PAC)	5 [2]	0.854 [0.089]	0.608 [0.218]	0.542 [0.184]	100%	408 [277]
	RCSI (entropy)	5 [2]	0.855 [0.092]	0.610 [0.223]	0.544 [0.184]	100%	435 [274]
	sharp score	5 [2]	0.868 [0.103]	0.642 [0.217]	0.579 [0.181]		5 [3]

Supplementary Table 1: Clustering performances of (consensus) hierarchical clustering with different calibration strategies from $N = 1{,}000$ simulated datasets corresponding to different levels of cluster separation. We simulate $N = 1{,}000$ datasets with $n = 150$ items split into $G^* = 5$ clusters such that $N_1 = 20$, $N_2 = 50$, $N_3 = 30$, $N_4 = 10$, $N_5 = 40$ across $p = 10$ features,

each with a proportion of explained variance of $E = 0.6$ (top), $E = 0.5$ (middle) or $E = 0.4$ (bottom). Median and inter-quartile range of the calibrated number of clusters (G), Rand index, Adjusted Rand Index (ARI), Jaccard index, and computation time in seconds for (consensus) hierarchical clustering with the simulated number of clusters (G^*), or calibrated by maximising the silhouette, Calinski–Harabasz (CH), Davies–Bouldin (DB) or GAP statistic. For consensus hierarchical clustering, we also report results when calibrating the number of clusters using the Δ, PAC, PINS discrepancy, RCSI and sharp scores. The RCSI is computed using $N = 25$ iterations. We also report the percentage of significant clustering structure for Monte Carlo approaches. For consensus clustering, the reported time includes both the computation of consensus matrices and the calibration procedure.

E	Method	G	Rand index	ARI	Jaccard index	Percentage	Time (s)
0.6	RCSI (PAC)	5 [1]	0.979 [0.049]	0.942 [0.128]	0.916 [0.169]	100%	642 [264]
	RCSI (entropy)	5 [1]	0.980 [0.038]	0.945 [0.100]	0.919 [0.135]	100%	708 [394]
0.5	RCSI (PAC)	5 [1]	0.936 [0.082]	0.824 [0.199]	0.765 [0.214]	100%	655 [234]
	RCSI (entropy)	5 [1]	0.939 [0.080]	0.832 [0.194]	0.774 [0.211]	100%	718 [298]
0.4	RCSI (PAC)	5 [2]	0.854 [0.090]	0.606 [0.219]	0.541 [0.187]	100%	808 [355]
	RCSI (entropy)	5 [2]	0.855 [0.091]	0.607 [0.222]	0.542 [0.186]	100%	814 [382]

Supplementary Table 2: Clustering performances of (consensus) hierarchical clustering calibrated by RCSI score using $N = 100$ iterations from $N = 1,000$ simulated datasets corresponding to different levels of cluster separation. We simulate $N = 1,000$ datasets with $n = 150$ items split into $G^* = 5$ clusters such that $N_1 = 20$, $N_2 = 50$, $N_3 = 30$, $N_4 = 10$, $N_5 = 40$ across $p = 10$ features, each with a proportion of explained variance of $E = 0.6$ (top), $E = 0.5$ (middle) or $E = 0.4$ (bottom). Median and inter-quartile range of the calibrated number of clusters (G), Rand index, Adjusted Rand Index (ARI), Jaccard index, and computation time in seconds, as well as the percentage of significant clustering structure are reported.

n	Method	G	Rand index	ARI	Jaccard index	Percentage	Time (s)
300	G*	5 [0]	0.924 [0.047]	0.792 [0.126]	0.727 [0.140]		0 [0]
	Silhouette	4 [2]	0.887 [0.204]	0.710 [0.370]	0.648 [0.303]		0 [0]
	CH	3 [2]	0.851 [0.197]	0.641 [0.334]	0.588 [0.259]		0 [0]
	DB	4 [2]	0.868 [0.242]	0.672 [0.422]	0.617 [0.331]		0 [0]
	GAP statistic	5 [0]	0.925 [0.039]	0.797 [0.109]	0.734 [0.123]		6 [3]
	G*	5 [0]	0.953 [0.034]	0.871 [0.091]	0.822 [0.113]		22 [15]
	Silhouette	4 [2]	0.921 [0.164]	0.796 [0.332]	0.737 [0.312]		22 [15]
	CH	3 [2]	0.872 [0.212]	0.697 [0.385]	0.643 [0.323]		22 [15]
	DB	4 [2]	0.914 [0.249]	0.780 [0.456]	0.722 [0.393]		22 [15]
	GAP statistic	10 [5]	0.876 [0.084]	0.608 [0.304]	0.513 [0.305]		22 [15]
	Delta	3 [1]	0.740 [0.129]	0.472 [0.200]	0.473 [0.136]		22 [15]
	PAC	20 [0]	0.768 [0.048]	0.318 [0.150]	0.309 [0.113]		22 [15]
	PINS discrepancy	20 [15]	0.772 [0.118]	0.338 [0.416]	0.330 [0.345]		22 [15]
	RCSI (PAC)	5 [1]	0.944 [0.076]	0.848 [0.179]	0.794 [0.196]	100%	1727 [1049]
	RCSI (entropy)	5 [1]	0.945 [0.071]	0.852 [0.168]	0.798 [0.184]	100%	2086 [1147]
	sharp score	5 [1]	0.946 [0.075]	0.855 [0.174]	0.802 [0.189]		22 [15]
600	G*	5 [0]	0.927 [0.036]	0.800 [0.100]	0.737 [0.112]		0 [0]
	Silhouette	4 [2]	0.876 [0.221]	0.697 [0.390]	0.638 [0.318]		0 [0]
	CH	3 [2]	0.849 [0.198]	0.640 [0.342]	0.591 [0.264]		0 [0]
	DB	4 [2]	0.859 [0.278]	0.663 [0.465]	0.612 [0.354]		0 [0]
	GAP statistic	5 [0]	0.927 [0.033]	0.804 [0.089]	0.742 [0.103]		27 [14]
	G*	5 [0]	0.949 [0.064]	0.862 [0.155]	0.811 [0.174]		65 [37]
	Silhouette	4 [3]	0.887 [0.243]	0.728 [0.449]	0.673 [0.388]		65 [37]
	CH	3 [2]	0.862 [0.216]	0.675 [0.387]	0.624 [0.322]		65 [37]
	DB	4 [3]	0.868 [0.338]	0.685 [0.564]	0.635 [0.451]		65 [37]
	GAP statistic	8 [3]	0.918 [0.062]	0.762 [0.206]	0.685 [0.236]		65 [37]
	Delta	3 [1]	0.730 [0.132]	0.457 [0.195]	0.465 [0.129]		65 [37]
	PAC	5 [15]	0.777 [0.223]	0.404 [0.570]	0.387 [0.496]		65 [37]
	PINS discrepancy	5 [15]	0.791 [0.229]	0.464 [0.573]	0.461 [0.499]		65 [37]
	RCSI (PAC)	5 [1]	0.929 [0.098]	0.813 [0.221]	0.756 [0.228]	100%	9924 [5464]
	RCSI (entropy)	5 [1]	0.931 [0.092]	0.819 [0.212]	0.762 [0.228]	100%	8479 [5505]
	sharp score	5 [2]	0.946 [0.117]	0.854 [0.252]	0.804 [0.257]		65 [37]

Supplementary Table 3: Clustering performances of (consensus) hierarchical clustering with different calibration strategies from $N = 1,000$ simulated datasets corresponding to different numbers of items. We simulate $N = 1,000$ datasets with $n = 300$ (top) or $n = 600$ (bottom) items split into $G^* = 5$ clusters such that $N_1 = 40$, $N_2 = 100$, $N_3 = 60$, $N_4 = 20$, $N_5 = 80$ (top) or $N_1 = 60$, $N_2 = 150$, $N_3 = 90$, $N_4 = 30$, $N_5 = 120$ (bottom) across $p = 10$ features, each with a proportion of explained variance of $E = 0.5$. Median and inter-quartile range of the calibrated number of clusters (G), Rand index, Adjusted Rand Index (ARI), Jaccard index, and computation time in seconds for (consensus) hierarchical clustering with the simulated number of clusters (G^*), or calibrated by maximising the silhouette, Calinski–Harabasz (CH), Davies–Bouldin (DB) or GAP statistic. For consensus hierarchical clustering, we also report results when cal-

ibrating the number of clusters using the Δ, PAC, PINS discrepancy, RCSI and sharp scores are reported. The RCSI is computed using $N = 25$ iterations. We also report the percentage of significant clustering structure for Monte Carlo approaches. For consensus clustering, the reported time includes both the computation of consensus matrices and the calibration procedure.

Approach	Method	G	q	Rand index	ARI	Jaccard index	Time (s)
	G*	5 [0]		0.779 [0.070]	0.403 [0.171]	0.381 [0.121]	0 [0]
	$\lambda_1 = 10.00$	5 [0]		0.820 [0.060]	0.490 [0.169]	0.436 [0.135]	120 [62]
	$\lambda_2 = 5.99$	5 [0]		0.832 [0.061]	0.522 [0.170]	0.462 [0.145]	120 [62]
	$\lambda_3 = 3.59$	5 [0]		0.842 [0.062]	0.552 [0.179]	0.486 [0.154]	120 [62]
	$\lambda_4 = 2.15$	5 [0]		0.849 [0.063]	0.572 [0.179]	0.504 [0.159]	120 [62]
Single run	$\lambda_5 = 1.29$	5 [0]		0.847 [0.062]	0.567 [0.178]	0.500 [0.154]	120 [62]
	$\lambda_6 = 0.77$	5 [0]		0.831 [0.059]	0.520 [0.166]	0.461 [0.139]	120 [62]
	$\lambda_7 = 0.46$	5 [0]		0.795 [0.058]	0.420 [0.164]	0.383 [0.123]	120 [62]
	$\lambda_8 = 0.28$	5 [0]		0.745 [0.051]	0.282 [0.141]	0.288 [0.091]	120 [62]
	$\lambda_9 = 0.17$	5 [0]		0.701 [0.037]	0.154 [0.098]	0.211 [0.055]	120 [62]
	$\lambda_1 0 = 0.10$	5 [0]		0.671 [0.021]	0.070 [0.055]	0.165 [0.029]	120 [62]
Consensus	G*	5 [0]		0.837 [0.095]	0.581 [0.216]	0.525 [0.178]	4 [2]
(unweighted)	sharp score	5 [2]		0.860 [0.086]	0.618 [0.202]	0.557 [0.179]	4 [2]
	$\lambda_1 = 10.00$	5 [0]		0.868 [0.084]	0.642 [0.205]	0.578 [0.187]	1761 [770]
	$\lambda_2 = 5.99$	5 [0]		0.879 [0.085]	0.673 [0.214]	0.602 [0.205]	1761 [770]
	$\lambda_3 = 3.59$	5 [0]		0.891 [0.083]	0.701 [0.210]	0.631 [0.205]	1761 [770]
	$\lambda_4 = 2.15$	5 [0]		0.895 [0.079]	0.710 [0.205]	0.638 [0.206]	1761 [770]
Consensus	$\lambda_5 = 1.29$	5 [0]		0.874 [0.081]	0.659 [0.204]	0.588 [0.193]	1761 [770]
(weighted)	$\lambda_6 = 0.77$	5 [0]		0.836 [0.086]	0.550 [0.210]	0.492 [0.169]	1761 [770]
	$\lambda_7 = 0.46$	5 [0]		0.758 [0.105]	0.360 [0.217]	0.355 [0.134]	1761 [770]
	$\lambda_8 = 0.28$	5 [0]		0.686 [0.095]	0.205 [0.166]	0.263 [0.080]	1761 [770]
	$\lambda_9 = 0.17$	5 [0]		0.630 [0.076]	0.094 [0.095]	0.209 [0.042]	1761 [770]
	$\lambda_1 0 = 0.10$	5 [0]		0.612 [0.061]	0.038 [0.055]	0.180 [0.025]	1761 [770]
	sharp score	5 [2]		0.923 [0.055]	0.787 [0.151]	0.718 [0.168]	1761 [770]

Supplementary Table 4: Comparison of clustering performances of (consensus) hierarchical clustering using the unweighted or COSA Euclidean distance. Performances are evaluated on $N = 1,000$ datasets with $n = 150$ items split into $G^* = 5$ clusters such that $N_1 = 20$, $N_2 = 50$, $N_3 = 30$, $N_4 = 10$, $N_5 = 40$ across $p = 100$ features, of which $q^* = 20$ have a nonzero proportion of explained variance ($E = 0.5$). Median and inter-quartile range of the calibrated number of clusters (G), Rand index, Adjusted Rand Index (ARI), Jaccard index, and computation time in seconds for hierarchical clustering with the simulated number of clusters (G^*) and different values of λ, consensus unweighted clustering with G^* or calibrated by the sharp score, and consensus COSA clustering with G^* and different values of λ or calibrated by maximising the sharp score are reported.
